# Maternal and birth cohort studies in the Gulf Cooperation Council countries: a systematic review and meta-analysis

**DOI:** 10.1186/s13643-020-1277-0

**Published:** 2020-01-16

**Authors:** Rami H. Al-Rifai, Nasloon Ali, Esther T. Barigye, Amal H. I. Al Haddad, Fatima Al-Maskari, Tom Loney, Luai A. Ahmed

**Affiliations:** 1 Institute of Public Health, College of Medicine and Health Sciences, United Arab Emirates University, P.O. Box 15551, Al Ain, United Arab Emirates; 2 College of Medicine, Mohammed Bin Rashid University of Medicine and Health Sciences, Dubai, United Arab Emirates

**Keywords:** Cohort studies, Infant health, Maternal exposure, Maternal health, Middle East, Prenatal exposure delayed effects, Review

## Abstract

**Background:**

We systematically reviewed and chronicled exposures and outcomes measured in the maternal and birth cohort studies in the Gulf Cooperation Council (GCC) countries and quantitatively summarized the weighted effect estimates between maternal obesity and (1) cesarean section (CS) and (2) fetal macrosomia.

**Methods:**

We searched MEDLINE-PubMed, Embase, Cochrane Library, Scopus, and Web of Science electronic databases up to 30 June 2019. We considered all maternal and birth cohort studies conducted in the six GCC countries (Bahrain, Kuwait, Oman, Qatar, Saudi Arabia, and United Arab Emirates (UAE)). We categorized cohort studies on the basis of the exposure(s) (anthropometric, environmental, medical, maternal/reproductive, perinatal, or socioeconomic) and outcome(s) (maternal or birth) being measured. Adjusted weighted effect estimates, in the form of relative risks, between maternal obesity and CS and fetal macrosomia were generated using a random-effects model.

**Results:**

Of 3502 citations, 81 published cohort studies were included. One cohort study was in Bahrain, eight in Kuwait, seven in Qatar, six in Oman, 52 in Saudi Arabia, and seven in the UAE. Majority of the exposures studied were maternal/reproductive (65.2%) or medical (39.5%). Birth and maternal outcomes were reported in 82.7% and in 74.1% of the cohort studies, respectively. In Saudi Arabia, babies born to obese women were at a higher risk of macrosomia (adjusted relative risk (aRR), 1.15; 95% confidence interval (CI), 1.10–1.20; *I*^*2*^ = 50%) or cesarean section (aRR, 1.21; 95% CI, 1.15–1.26; *I*^*2*^ = 62.0%). Several cohort studies were only descriptive without reporting the magnitude of the effect estimate between the assessed exposures and outcomes.

**Conclusions:**

Cohort studies in the GCC have predominantly focused on reproductive and medical exposures. Obese pregnant women are at an increased risk of undergoing CS delivery or macrosomic births. Longer-term studies that explore a wider range of environmental and biological exposures and outcomes relevant to the GCC region are needed.

**Systematic review registration:**

PROSPERO CRD42017068910

## Background

A wide range of prenatal exposures including environmental, genetic, and socioeconomic factors can individually or jointly affect different maternal and birth health outcomes [1–3]. Such unfavorable health outcomes might manifest during the early or later stages of pregnancy or infancy leading to both short- and long-term consequences [1–3]. For instance, gestational diabetes mellitus (GDM) increases the risk of both the mother developing post-pregnancy type 2 diabetes mellitus (T2DM) [4] and macrosomia in the newborn [5]. Maternal obesity has also been associated with an increased risk of macrosomia in newborns [5]. Socioeconomic exposures including poverty and environmental factors, such as air pollution, have also been shown to be associated with various maternal and birth outcomes [6–9]. Pre-eclampsia is positively associated with a greater risk of developing cardiovascular diseases (CVD) or cardiac shock in the future [10–12], and it doubles the risk of stroke in the offspring [13].

High-quality and well-designed cohort studies provide robust data that can be used to explore associations between specific exposures and outcomes. Long-term birth cohort studies such as the Norwegian Mother and Child Study [14] and the Danish Birth Cohort Study [15] have revealed several important maternal and child factors operating in early life, fetal growth, and its determinants. However, the information obtained in these settings may not be easily generalized to different populations, such as the Gulf Cooperation Council (GCC) countries (i.e., Bahrain, Kuwait, Oman, Qatar, Saudi Arabia, and the United Arab Emirates (UAE)), as they may have different individual, familial, lifestyle, environmental, and genetic exposures including, but not limited to consanguinity, physical inactivity, diet, and tobacco use.

In recent decades, there has been a dramatic rise in the prevalence of several adverse health outcomes in the GCC countries, in particular non-communicable diseases and their risk factors including obesity, T2DM, asthma, neurodevelopmental disorders, and CVD [16, 17]. Maternal and prenatal exposures and associated outcomes in these GCC countries have become of great interest due to changes in demographic dynamics, composition of the population, and lifestyle transition [16]. Among females, the prevalence of physical inactivity is very high (58.7–98.7%) and the proportion of women that report smoking cigarettes or water pipes varies considerably (0.5–20.7%) [18].

There are number of cohort studies that have been conducted in the GCC countries that pertain to specific exposures and outcomes affecting maternal and infant health [19–22]. These include anthropometric, environmental, socioeconomic, lifestyle, and medical physiological exposures that can bear consequences on the pregnancy condition, delivery process, neonatal status, perinatal growth, and possibly long-term health consequences for both the mother and offspring [23–27]. However, there has not been a synthesis and evaluation of the different cohort studies that have been conducted in the GCC countries on which to base more effective evidence-based public health policies. A comprehensive review of the maternal and birth cohort literature in the GCC will highlight research areas that have received considerable attention and identify knowledge gaps in the current body of scientific evidence. Highlighting understudied maternal and child health-related exposures and outcomes is important for grant funding bodies tasked with identifying priority areas and researchers planning future studies.

The objectives of this study are (i) to summarize and characterize the exposures and outcomes that have been examined and discussed in the maternal and birth cohort studies in the six GCC countries (qualitative synthesis) and (ii) to quantitatively generate weighted effect estimates on the association between maternal obesity and (a) cesarean section (CS) and (b) fetal macrosomia (quantitative synthesis).

## Materials and methods

The protocol for this review has been published elsewhere [28] and is registered online on PROSPERO (registration number CRD42017068910). Minor necessary modifications not in line with the protocol were adapted in this review, whenever it was necessary. Our review was informed by the Cochrane Collaboration guidelines [29] and reported according to the Preferred Reporting Items for Systematic Reviews and Meta-Analyses (PRISMA) guidelines [30]. The PRISMA checklist can be found in (see Additional file 1: Table S1).

### Data source and search strategy

We searched MEDLINE-PubMed, EMBASE, Scopus, Web of Science, and The Cochrane Library databases (up to 30 June 2019). We used comprehensive search criteria with no language restrictions. The literature search protocol is summarized in the (see Additional file 2: Box S1).

### Study selection

Retrieved citations from the six databases were imported and compiled into EndNote reference manager [31], and duplicate records were removed. The remaining records were reviewed at the title/abstract level, and full texts of those records that were considered eligible or potentially eligible against our eligibility criteria were retrieved for full-text review. In this review, we use the term “cohort study” to refer to a full published research article containing a followed up maternal and/or birth cohort(s).

**Fig. 1:**
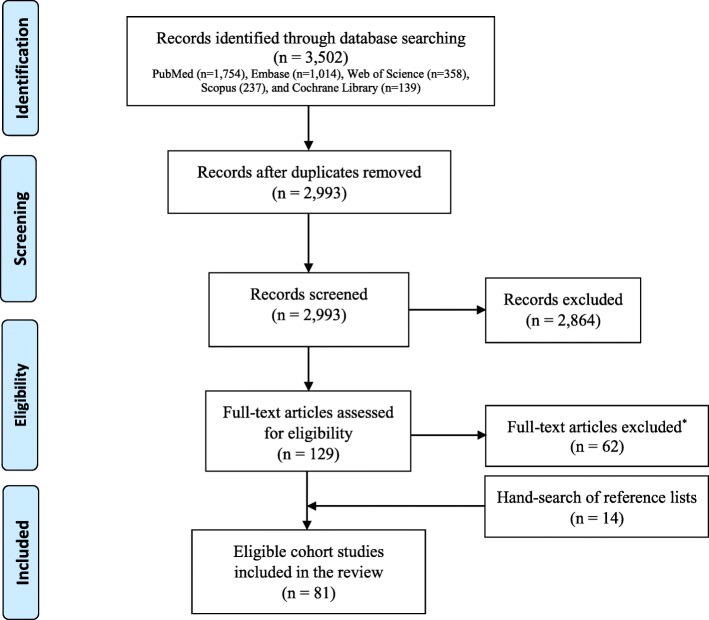
PRISMA flow diagram of article selection process

Two reviewers (NA and ETB) independently assessed retrieved citations for eligibility. Full-text articles deemed relevant or potentially relevant were retrieved and screened against specific inclusion and exclusion criteria. We also systematically screened the reference lists of all eligible cohort studies for further eligible publications (Fig. 1). Conflicts were resolved by discussion and consensus after consulting expert reviewers (RHA and LA).

### Eligibility criteria

#### Inclusion criteria

- Study design: prospective or retrospective cohort.
- Study population: pregnant mothers and their offspring.
- Geographical location: any of the six GCC countries, namely, Bahrain, Kuwait, Oman, Qatar, Saudi Arabia, or the UAE.
- Recruitment timing. Cohort studies should have recruited pregnant mothers and their newborns or recruited newborns immediately after delivery as long as relevant information on pregnancy was available.
- Follow-up: no specific follow-up period. Cohort studies were required to have some prospective or retrospective data on exposure(s) and outcome(s) for mothers and/or their offspring.
- Measurements: not specific. Cohort studies should have measured at least one maternal exposure and at least one maternal and/or newborn outcome.

#### Exclusion criteria

We excluded all other study designs including cross-sectional, case-control, randomized-controlled trials, reviews, qualitative studies, editorials, author commentaries, and case series studies regardless of the number of cases. Studies that were not conducted in GCC countries or were not attributed to any of the GCC countries were also excluded.

#### Data extraction and management

We extracted and summarized data following the PECO framework [32, 33]. The PECO stands for population, exposure, comparator, and outcome. Summarizing cohort studies using the PECO framework helps to identify the specific population, exposure(s), and outcomes(s) assessed in the cohort(s), being followed.

Data were extracted using a pre-piloted data extraction form. Data extraction was performed by two reviewers (NA and ETB). Random checks of at least 50% of the extracted studies were crosschecked by a third expert reviewer (RHA). Discrepancies in data extraction were resolved by agreement between data extractors and the third expert reviewer (RHA). The main features of the eligible cohort studies including author name(s), year of publication, and study design were extracted. In addition, we also extracted different characteristics of the studied population including recruited cohort population, country, size of the cohort, measured exposure(s) and outcome(s), and key findings of the cohort study, whenever reported and possible. If reported, we also extracted adjusted effect estimates of the association between maternal obesity and CS or macrosomia. In studies adjusting the effect estimate using different models, we extracted estimates from the model that we considered to adjust for the most appropriate confounders (e.g., age, parity, comorbidity). If the cohort study had discrepant effect estimates in the text and tables, we extracted estimates reported in text if the corresponding author(s) of these studies failed to respond to our email enquiries.

In this review, all factors or variables treated in the original cohorts as risk factors or independent/explanatory variables that could be determinant of health outcome(s) of interest were defined as exposures. According to the nature and source, we categorized the defined exposures into six exposure domains (anthropometric, environmental, medical/medical service, maternal/reproductive, perinatal/infant, and sociodemographic) and the measured outcomes into two outcome domains (birth and maternal outcomes). Hence, one cohort study could incorporate more than one exposure domain and/or more than one outcome domain. Indeed, one of the strengths of the cohort study design is the capability to assess multiple exposures and outcomes in the same cohort. Anthropometric domain included measures such as height, body mass, and body mass index (BMI) of the mother. Environmental domain included, for example, living conditions, nutritional exposures, and exposure to any type of smoke. Medical/medical service domain referred to pre-existing maternal health conditions that were not due to pregnancy including medical conditions such as DM or hypertension as well as a family history of diseases and/or medical services during pregnancy and delivery such as length of waiting time to receive healthcare, relationships with the healthcare professionals, consumption of medication, or depression. Maternal/reproductive domain referred to conditions that were specifically related to exposures experienced during present or previous pregnancies or post-delivery such as parity, GDM, or breastfeeding. Perinatal/infant domain included exposures, for example, birth weight, multiple birth, and mode of birth delivery. Sociodemographic domain included exposures such as age, education, or employment.

In this review, all variables or measures treated in the original cohorts as dependent variables that may stem from exposure to potential risk factor(s)/independent variable(s) were defined as outcomes. The defined outcomes were categorized into two domains (maternal or birth) indicating whether the mother or her newborn suffered from an outcome due to a specific exposure.

#### Risk of bias assessment

We evaluated the methodological quality and risk of bias (ROB) aspects for each cohort study using the National Heart, Lung, and Blood Institute (NIH) tool [34]. For each assessed criteria, each study has the potential to be categorized as “potentially of low ROB” if the answer was “yes” for that specific criteria, “potentially of high ROB” if the answer was “no” for that specific criteria, or “can’t determine, not applicable, or not reported” for that specific criteria. ROB was performed by at least two reviewers for each study.

### Quantitative analysis

#### Meta-analysis

Meta-analyses of pre-calculated adjusted effect estimates were conducted, and the corresponding 95% confidence interval (CI) was estimated. We pooled adjusted estimates using a random-effects model [35]. We estimated the I-squared (*I*^*2*^) as a measure of heterogeneity [36, 37]. Meta-analyses were performed using the Review Manager (RevMan) version 5.3 [38]. In cohort studies reporting adjusted odds ratio (aOR) as a measure of effect estimate, we converted the aOR into adjusted relative risk (aRR) following a standard procedure [39]. Odds ratio (OR) are not well understood, and when the outcome is common, OR are always further away from 1 than relative risk (RR). Misinterpretation of the OR in cohort studies can potentially lead to serious overestimation of the effect estimate between an exposure and outcome being studied [39].

### Ethics approval

In line with the United Arab Emirates University-Human Research Ethics Committee regulations, ethical approval or an exemption letter was not required for this study as it did not use any primary data.

## Results

### Scope of the review

We identified 3502 citations. Of which, 81 citations were found eligible as maternal and birth cohort studies for inclusion in the systematic review (Fig. 1).

### Study characteristics

Table 1 summaries the 81 published cohort studies according to the measured six exposures and two outcomes domains in the six GCC countries. Additional file 3: Table S2 presents more information on the measured exposures and outcomes in addition to the summary of key findings of each of the reviewed 81 cohort studies in the GCC countries, stratified by the country.

**Table 1:** Summary of the reviewed 81 published cohort studies according to the measured six exposure and two outcome domains

Measured	Number of cohorts studies^a^ [Ref]	Percentage out of the 81 research reports
Exposures		
Anthropometric (e.g., BMI)	16 [40–55]	19.8
Environmental (e.g., nutrients, smoking)	6 [49, 51, 56–59]	7.4
Medical/Medical services (e.g., non-maternal diseases, hospital stay)	32 [20, 21, 26, 27, 40, 41, 43–46, 52, 53, 58, 60–78]	39.5
Maternal or reproductive (e.g., parity, GDM)	52 [22, 24, 25, 40, 41, 43–46, 48, 52, 53, 56, 58, 61, 63–68, 71, 72, 74–76, 79–104]	65.2
Perinatal or newborn (e.g., birth weight, cord blood)	17 [20, 21, 43, 45, 46, 53, 65, 69, 73, 75, 92, 105–110]	21.0
Sociodemographic (e.g., age, income)	25 [24, 40, 41, 43–46, 51–53, 56, 58, 75, 76, 84, 87, 92, 99, 100, 102, 104, 110–113]	30.9
Outcomes		
Maternal (e.g., C-section, pre-eclampsia)	60 [22, 24–26, 40–48, 50, 53–56, 58–62, 64, 66–68, 71, 72, 74, 76–91, 93–100, 102–104, 111–113]	74.1
Birth (e.g., macrosomia, stillbirth)	67 [20–22, 25–27, 40, 42, 43, 45–52, 54–57, 60–63, 65–75, 77–80, 82, 85–98, 101–103, 105–113]	82.7

*BMI* body mass index, *GDM* gestational diabetes mellitus, *C-section* cesarean section

^a^Some cohorts measured multiple exposures and multiple outcomes

The 81 cohort studies were published between 1990 in Saudi Arabia [40, 79] and 2019 in Kuwait [41], Qatar [42, 60, 61], and Saudi Arabia [80]. The size of the cohorts ranged from 23 pregnant women with a known diagnosis of idiopathic thrombocytopenic in Saudi Arabia [62] to 158,006 delivering mothers in Kuwait [81]. Majority (64.2%) of the cohort studies were in Saudi Arabia [25–27, 40, 43–50, 56, 57, 62–72, 79, 80, 82–98, 105–112], followed by eight (9.9%) in Kuwait [20, 21, 24, 41, 51, 73, 81, 99], seven (8.6%) in each of Qatar [42, 52, 53, 60, 61, 74, 113] and the UAE [58, 59, 75–77, 100, 101], six (7.4%) in Oman [54, 55, 78, 102–104], and one (1.2%) in Bahrain [22] (see Additional file 3: Table S2).

Thirty-four cohort studies (42.0%) were identified as a prospective design (19 in Saudi Arabia, seven in Kuwait, four in UAE, two in Qatar, one in Oman, and one in Bahrain) [20–22, 24, 40, 41, 45, 47, 50, 51, 53, 55, 57–59, 63–65, 73, 76, 79, 85, 92, 94, 97, 99, 100, 106–109, 111, 113] while 47 (58.0%) used a retrospective design [25–27, 42–44, 46, 48, 49, 52, 54, 56, 60–62, 66–69, 71, 72, 74, 75, 77, 78, 80–84, 86–91, 93, 95, 96, 98, 101–105, 110, 112] (33 in Saudi Arabia, five in Qatar, five in Oman, one in Kuwait [81], and three in the UAE). Fifty-two (64.2%) cohort studies enrolled pregnant mothers at varying stages of their pregnancy with different characteristics such as diabetic and non-diabetic mothers [26, 67], obesity [42, 47, 54], singleton [41, 48, 99] or triplet pregnancies [91], teenage women [112], multipara women [82, 86, 98], and women with systemic lupus erythematosus (SLE) [27]. Eight studies (9.9%) enrolled pregnant mothers at varying stages after delivery [24, 49, 58, 59, 81, 84, 88, 99]. Seventeen studies (21.0%) recruited newborns at varying stages after birth [20, 21, 45, 52, 56, 63, 65, 66, 70, 73, 75, 92, 101, 102, 105–108] such as preterm babies [20, 66, 73, 105, 108] (see Additional file 3: Table S2).

### Studied exposures

Majority (65.2%) of the 81 cohort studies discussed maternal or reproductive exposures followed by medical/medical service exposures (39.5%) and sociodemographic exposures (30.9%) (Table 1).

#### Maternal or reproductive exposures

Maternal or reproductive exposures often measured were GDM and parity in 24.7% and 16.0% of the 81 cohort studies, respectively. GDM was investigated as an exposure for different maternal and birth outcomes including mode of birth delivery, birth weight, APGAR score, preterm delivery, intrauterine fetal death, and admission to neonatal intensive care unit (NICU) in four cohort studies [26, 48, 67, 88]. These cohorts consistently found that pregnant women with pre-GDM or GDM were at increased risk of various adverse maternal and birth outcomes including CS delivery, macrosomia, and preterm delivery [26, 48, 67, 88]. Pre-GDM was also independently associated with CS delivery (adjusted odds ratio “aOR,” 1.65), induction of labor (aOR, 1.67), macrosomia (aOR, 1.40), stillbirth (aOR, 3.66), and APGAR score < 7 at 5 min (aOR, 3.82) [67]. Various unfavorable health outcomes were more common in grand multipara compared to primigravida mothers [82, 114] (see Additional file 3: Table S2).

#### Sociodemographic exposures

Maternal age was a common measured sociodemographic exposure studied in 18 cohort studies. Advanced maternal age was associated with GDM, CS, and preterm delivery [51, 53, 58, 92, 111, 113]. Primary education or less was independently associated with 69% lower likelihood of exclusive breastfeeding at 6 months (aOR, 0.31; 95% CI, 0.11–0.88) [58] (see Additional file 3: Table S2).

#### Medical or medical service exposures

Thirty-two (39.5%) cohort studies explored medical or medical services as exposures such as length of hospital stay [58] and other medical conditions such as SLE [27, 102] and diabetes [26, 60, 67, 68, 70, 74]. Pre–pregnancy T1DM or T2DM were independently associated with emergency (aOR, 2.67) or elective CS delivery (aOR, 6.73), macrosomia (aOR, 3.97), or preterm delivery at < 37 weeks (aOR, 2.24) in Saudi Arabia [26].

#### Other exposures

Seventeen (21.0%) cohort studies focused on perinatal exposures. These included factors such as birth weight [20, 21, 73, 106], head circumference [92], and birth multiplicity [93]. Environmental and anthropometric exposures were measured in only six (7.4%) and 16 (19.8%) of the 81 cohort studies, respectively. Environmental exposures included smoking and secondhand smoking [49, 56], and all six studies on anthropometric measures were on BMI (Table 1 and see Additional file 3: Table S2).

### Studied outcomes

There were 21 cohort studies reporting only birth outcomes [20, 21, 27, 49, 51, 52, 57, 63, 65, 69, 70, 73, 75, 92, 101, 105–110], 14 cohort studies reporting only maternal outcomes [24, 41, 44, 53, 58, 59, 64, 76, 81, 83, 84, 99, 100, 104], and 46 cohort studies reporting both maternal and birth outcomes [22, 25, 26, 40, 42, 43, 45–48, 54–56, 60–62, 66, 68, 71, 72, 74, 77–80, 82, 85–91, 93–98, 101–103, 111–113] (Table 1 and, see Additional file 3: Table S2).

#### Maternal outcomes

Mode/type of birth delivery assessed in 15 cohort studies [14, 40, 47, 48, 50, 55, 62, 67, 68, 71, 72, 81, 86, 88, 111], followed by preeclampsia/eclampsia in 12 cohorts [47, 50, 53, 54, 68, 72, 93, 97, 102, 103, 111, 113], GDM in seven cohort studies [47, 50, 53, 54, 83, 111, 113], and maternal anemia in three cohort studies [50, 104, 113]. Postpartum depression was explored in only one prospective cohort study in the UAE [76]. Pregnancy anemia was examined in only one cohort in Oman [104]. In several cohort studies, obese pregnant women were at a higher risk of developing several unfavorable outcomes including GDM (aOR, 5.10 [47]; aOR, 6.60 [53]; RRs, 8.60 [50]), pregnancy hypertension (RRs, 6.10 [50]; RRs, 6.10 [50]; aOR, 2.23 [47]), pre-eclamptic toxemia (RRs, 5.90 [50]), CS delivery (aOR, 4.80 [47]; aOR, 2.16 [48]; RRs, 2.00 [50]), antepartum (aOR, 2.80) or postpartum hemorrhage (RRs, 2.50) [47], macrosomia (aOR, 6.80 [50], 9.18 [49], 3.90 [47]), 1 min APGAR score < 7 (RRs, 6.80) [50], postdate delivery (> 42 weeks) (RRs, 3.70) [50], and preterm birth (aOR, 2.20) [47]. Obese pregnant women with GDM (aOR, 3.45) or obese pregnant women with no GDM (aOR, 1.46) were more likely to deliver macrosomic babies compared to non-obese pregnant women with no GDM [48].

#### Birth outcomes

The most common measured birth outcome was birth weight in 33 cohort studies [22, 26, 40, 42, 47–51, 54–56, 67, 68, 70–72, 75, 78–80, 85, 86, 88–90, 96–98, 101–103, 111], followed by congenital malformations in nine cohort studies [22, 43, 54, 57, 68, 70, 78, 97, 113], preterm birth in 12 cohort studies [51, 67, 70, 71, 77, 79, 88, 90, 93, 97, 103, 111], and stillbirth in five cohort studies [22, 25, 51, 110, 111]. Retinopathy of prematurity was assessed in three preterm birth cohorts in Kuwait [20, 21, 73]. Early cognitive development of infants at different early life stages was explored in only one cohort [109], and mean umbilical cord blood lead level was also measured in one other cohort [92]. Maternal, fetal, or neonatal deaths were examined in 12 cohort studies [25, 26, 62, 63, 68, 82, 85, 87, 90, 93, 112]. Eczema in children at 2 years of age was assessed in only one cohort in Saudi Arabia that linked to the sub-optimal growth indexed by fetal abdominal circumference [69] (see Additional file 3: Table S2).

### Weighted effect estimates

**Fig. 2:**
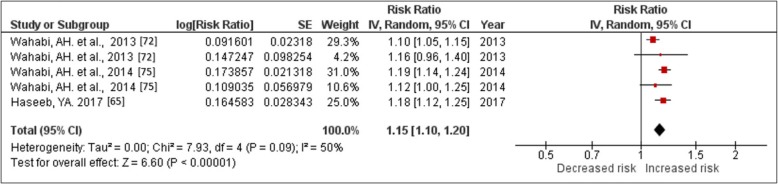
Pooled adjusted estimates of the association between maternal obesity and macrosomia. Note: Estimates from same author and year indicates to stratified estimates that were extracted from same study and included in the forest plot. Square indicates to the study-specific effect estimate. Size of the square is proportional to the precision (weight) of the study-specific effect estimates. Bars indicate the width of the corresponding 95% confidence interval (CI). The diamond centered on the summary effect estimate, and the width indicates the corresponding 95% CI

**Fig. 3:**
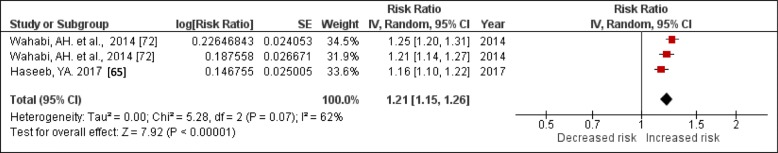
Pooled adjusted estimates of the association between maternal obesity and CS delivery. Note: Estimates from same author and year indicates to stratified estimates that were extracted from same study and included in the forest plot. Square indicates to the study-specific effect estimate. Size of the square is proportional to the precision (weight) of the study-specific effect estimates. Bars indicate the width of the corresponding 95% confidence interval (CI). The diamond centered on the summary effect estimate, and the width indicates the corresponding 95% CI

Obese pregnant women in Saudi Arabia were 15% more likely to give birth to a macrosomic baby compared to non-obese women (pooled aRRs, 1.15; 95% CI, 1.00–1.25; *I*^*2*^ = 50.0%) (Fig. 2) [47–49]. Following written communication with the study authors [48], we excluded two unverified point estimates due to the inaccuracy of the reported CI. Nonetheless, Saudi obese pregnant women remained at a higher risk of giving birth to a macrosomic baby compared to non-obese mothers (pooled aRR, 1.18; 95% CI, 1.14–1.22; *I*^*2*^ = 0.0%) (see Additional file 4: Figure S1). Obese pregnant Saudi women were also at a 21% increased risk of undergoing CS delivery compared to non-obese pregnant Saudi women (pooled aRRs, 1.21; 95% CI, 1.15–1.26; *I*^*2*^ = 62.0%) (Fig. 3). Excluding one estimate (aOR, 4.80; 95% CI, 1.50–6.40), due to the inability to verify the accuracy of the reported CI following written communication with the study authors [47], did not change the strength of this association (aRRs, 1.23; 95% CI, 1.19–1.28; *I*^2^, 15%) (see Additional file 5: Figure S2).

### Quality assessment

Findings of our summarized and criteria-specific quality assessment of cohort studies can be found in the supplementary information. Briefly, all studies clearly stated the research question(s)/objective(s), clearly specified and defined the study population, and recruited subjects from the same or similar populations with stating the inclusion and exclusion criteria. Hence, all cohort studies were categorized as “potentially of low ROB” for these three assessment criteria. Over a half (57.0%) of the cohort studies either reported descriptive statistics for the burden of the exposure and outcomes or the association between the measured exposure(s) and outcome(s) was not adjusted for any potential confounding effect, and hence were classified as “potentially of high ROB”. Overall, cohort studies were of reasonable quality with “potentially of low ROB” in 9.8 and with “potentially of high ROB” in 1.6 of the 14 measured quality criteria (see Additional file 6: Figure S3, and, see Additional file 7: Table S3).

## Discussions

### Summary of major findings

The present systematic review summarizes the published evidence on the maternal and birth cohort studies that have been conducted in the six GCC countries. This is the first review to chronicle, synthesize, and appraise the maternal and birth cohort studies in the GCC countries. The review confirms, using peer-reviewed data, that pregnant women in the GCC countries have a high burden of various maternal and modifiable lifestyle and environmental exposures. These exposures were associated with a range of different unfavorable maternal and birth health-related outcomes. Saudi Arabia contributed the largest volume of literature to the review. The included cohort studies predominantly focused on maternal and reproductive exposures compared to other aspects of pregnancy such as the biological predisposition of the mother or the type of environment. Majority of the studies reported only descriptive estimates on the burden of exposures and/or outcomes or crude estimates on the association between exposure(s) and outcome(s). Our summary effect estimates strengthened the evidence base for a strong positive association between maternal obesity and macrosomia or CS delivery.

### Implications for clinicians and policy makers

Globally, the prevalence of obesity has nearly tripled since 1975 [115]. The populations in the GCC countries have also been affected by this global trend in overweight and obesity. According to the World Health Organization (WHO) report in 2010, the prevalence of obesity in females in Kuwait was 48%, 44% in Saudi Arabia, 42% in the UAE, 38% in Bahrain, 32% in Qatar, and 17% in Oman [116]. A recent report issued by the WHO in 2017 documented that the age standardized prevalence of obesity in females in each of the six GCC countries exceeded 30% [117]. Our meta-analyses revealed that maternal obesity is independently positively associated with undergoing CS delivery or giving birth to a macrosomic baby. Our weighted estimate showed that obese women were 1.15-times more likely to have macrosomic babies (Fig. 3) which is similar to the pooled estimate from 16 case-control and cohort studies in Asia (India), Europe (Denmark, France, Germany, Italy, and the UK), the Middle East (Saudi Arabia), and North America (Canada, USA) (RRs, 1.20; 95% CI, 1.18–1.23) [118]. Also, maternal obesity increases the risk of GDM which is also a risk factor for different drivers of CS delivery including congenital disorders and anticipated low birth weight [70] and macrosomia and dystocia [67]. However, two of the three estimates included in our overall pooled estimate on the association between obesity and CS adjusted for GDM, maternal age, parity, gestational age, and exposure to environmental tobacco smoke [48]. Our weighted effect estimate on the maternal obesity and CS delivery (Fig. 3) is similar to previously published weighted estimates reported (1) in 2008 in 11 cohort studies in different European countries and the USA (RR, 1.21; 95% CI, 1.18–1.22) [119]; (2) in 2007 in 33 cohort studies in USA, Sweden, France, Denmark, Israel, Canada, the UK, Poland, and the United Arab Emirates (RR, 1.20; 95% CI, 1.18–1.22) [120]; and in 2015 in 22 cohort studies in low and middle-income countries in Southeast Asia, Middle East, and Central and South America (RR, 1.19; 95% CI, 1.10–1.26) [121]. Our weighted estimate was also similar to the previously reported weighted estimate (RR, 1.20; 95% CI, 1.16–1.23) on the association between severe maternal obesity and CS delivery reported in 33 cohort studies in 2007 [120]. This finding from studies in GCC countries strengthens the evidence base on the strong positive association between maternal obesity and undergoing CS delivery even after adjustment for maternal age and parity. Previous research has reported that if normal weight women have a 20% increased risk of CS delivery while obese women have a 40% increased risk of CS delivery, then every 1% decrease in the fraction of obese pregnant women would prevent 16,000 CS deliveries annually [120]. Hence, with our documented 21% increase risk of CS in obese women in the GCC countries, a substantial number of CS deliveries and related complications would be averted when reducing the prevalence of maternal obesity in these countries.

### Gaps in evidence

Many of the cohort studies followed specific subpopulations rather than a representative population of the country. Nearly all cohorts were recruited through either convenience or consecutive sampling. The majority of studies only followed the cohorts for a short period of time (e.g., third trimester to birth). As such, larger population-based studies with longer follow-up periods are needed to further understand the long-term influence of early and late prenatal exposures on pregnancy outcomes as well as on health and developmental outcomes during infancy, early childhood, adolescence, and adulthood.

This review has shown that there is a lack of research exploring the relationship between environmental exposures and maternal and child health outcomes. This is problematic for several reasons. In the GCC, rapid modernization has occurred over the last 50 years which might have had detrimental effects on the environment [122]. Factors such as water pipe smoking and indoor incense use are prevalent and have been shown to increase the risk of several adverse maternal outcomes such as wheezing, asthma, and headache, which in turn may affect the developing fetus [123].

Furthermore, consanguinity is prevalent in the GCC region, which increases the risk of genetic-related health outcomes such as thalassemia [124] that can negatively impact pregnancy and child birth [125]. However, early intervention and management can minimize the negative consequences of these conditions on the delivery of the child and their prognosis.

The lack of the above exposures being studied leaves a gap in the literature on how a multitude of exposures which are not sociodemographic or reproductive in nature may affect the health and lives of the mother and offspring. Longitudinal prospective cohorts collecting a large and varied dataset are relevant and necessary to understand this knowledge gap.

9With respect to outcomes, longitudinal maternal and child cohort studies should endeavor to measure a broad range of health conditions. This has been discussed by Golding [126] who draws parallels between successful cohorts around the world such as the Avon Longitudinal Study of Pregnancy and Childhood (ALSPAC) cohort [126], the Danish National Birth Cohort (DNBC) [15], and the Norwegian Mother and Child Cohort Study (MoBa) [127]. Golding stipulates the studied outcomes should include parental, pregnancy-based, and baby- or child-based outcomes including anthropometric, signs and symptoms of illness during childhood, and behavioral and mental health measures in children and adolescents [126]. Such studies require a longer follow-up than the previously conducted mother and child cohort studies in GCC countries.

### Implications for improved reporting and interpretation of future cohort studies

Cohort studies aim to identify risk factors leading to the development of unfavorable specific health outcomes. Designing and implementing cohort studies is a time- and effort-consuming process. Reporting results of cohort studies should follow a robust methodology and should be informative using appropriate scientific terms as well as appropriate bio-statistical analyses. In many of the reviewed cohort studies, there were weaknesses in appropriately reporting the correct study design, using the appropriate epidemiologic terms, or implementing the appropriate bio-statistical analyses. Cohort studies are not the same as case-control or cross-sectional studies [128]. “Incidence rate and incidence proportion” are different from “prevalence” [129]. Measuring strength and the magnitude of association between the measured exposure(s) and the outcome(s) after controlling for the influence of potential confounders is critical. Limiting the reporting to descriptive crude results in the form of proportions or correlations is not sufficient. What should we measure, OR or RRs? In medical research, there is a confusion on interpreting OR [39]. The OR is usually further away from 1 than the RR except in rare outcomes [39]. Misinterpretation of the OR can lead to serious overestimation of the benefits or risks in medical decision-making that may confuse healthcare professionals and policy makers, discussing treatment options or designing public health interventions [39]. Odds ratio is not well understood as a measure of effect size, and conversion to RRs by a simple calculation would improve understanding of findings [39]. When communicating results of medical research, it is important to be able to frame the statistics in a meaningful and easily understood metric [130]. Quantifying RR as a metric of the effect size is more appropriate and informative [39]. In the reviewed cohort studies, even in studies that went beyond descriptive analysis, researchers relied mainly on estimating the OR rather than the RR. Clinician researchers without a background in cohort methodology should involve epidemiologists in the design, conduct, analysis, and reporting of cohort studies as this would improve the quality and interpretation of the available evidence.

### Strengths and limitations

To our knowledge, this is the first review to explore the types of exposures and outcomes being studied in maternal and birth cohorts in the GCC region. We implemented a comprehensive search strategy covering four electronic databases in addition to hand-searching of reference lists of included studies. We carefully screened studies and extracted data and critically assessed the ROB of the included cohort studies using the National Institute of Health scale (see Additional file 7: Table S3). Consequently, our paper represents a comprehensive review mapping gaps in evidence and provides critical recommendations to improve analyzing and reporting results of cohort studies.

Some limitations should be considered when interpreting the findings of this review. First, the review was limited to the available adjusted effect estimates from a narrow range of specific exposure-outcome pairs from Saudi Arabia only. This has also limited our ability to quantify the sources of heterogeneity through meta-regression and subgroup analyses. Secondly, there were inherent differences in the designs of these cohorts and measurement methods of even similar exposures and outcomes which may account for some of the observed small-to-moderate heterogeneity (*I*^*2*^ = 15–62%) and could affect the strength of evidence from our meta-analyses. The results of our meta-analyses provide supporting evidence on the association between maternal obesity and fetal macrosomia or CS. However, careful consideration should be given when interpreting findings as some of the individual point estimates included in our meta-analyses might be biased. These individual point estimates were based on a varied cutoff point used to identify the exposed population. For example, the WHO defines obese people as those with a BMI ≥ 30 kg/m^2^ [131]; however, in one study which provided two adjusted estimates in the meta-analyses, obese women were defined as having a BMI ≥ 29.9 kg/m^2^ [47]. Using a slightly lower BMI cutoff to classify obesity may have overestimated the burden of the exposed population leading to misclassification bias. Residual confounding bias is also a potential limitation when interpreting any reported associations. Thirdly, as we did not search national databases, there is a limited possibility that we might have missed some unpublished maternal and birth cohort studies conducted in the GCC countries. However, this limited possibility is (i) supported by the robust searching and screening strategy we implemented and (ii) the lower likelihood that well-conducted cohort studies would not be published in indexed peer-reviewed journals.

Studies limited the recruited cohort to only citizens which minimizes the generalizability of the findings to the general population. For instance, our pooled estimates on CS and macrosomia were limited to the Saudi population which reduces the representativeness to the other five GCC countries or to other nationalities living in Saudi Arabia. Overall, this does not detract from the importance of our meta-analysis findings (Fig. 2, see Additional file 4: Figure S1, and Fig. 3, see Additional file 5: Figure S2) which are consistent with previously published findings from populations outside the GCC [118–120].

Despite these limitations, our review compiled and summarized important data and provided narrative information from a large number of maternal and birth cohort studies in the six GCC countries. Our review was also able to provide specific weighted estimates in Saudi Arabia, the largest of the six GCC countries in terms of population size and land mass.

## Conclusions

The reviewed maternal and birth cohort studies in the GCC countries have focused on reproductive and sociodemographic exposures. Birth outcomes were studied more frequently than maternal outcomes. Obese pregnant women are at higher risk of undergoing CS delivery or giving birth to macrosomic babies. Designing future cohort studies should strive to explore a wide range of mother and child exposure outcomes that are relatively under-researched but prevalent in the GCC countries such as various forms of tobacco use and air quality, DM and GDM, and consanguinity. These future studies will provide informative data to fill gaps in the evidence. Such findings can be used by clinicians, researchers, and policy makers to address important maternal and child health issues.

## Supplementary information

**Additional file 1: Table S1.** Preferred Reporting Items for Systematic Reviews and Meta-analyses (PRISMA) 2009 checklist [30].

**Additional file 2: Box S1**. Data sources and search criteria for systematically reviewing literature reporting on maternal and birth cohort studies in the GCC Countries.

**Additional file 3: Table S2**. Summary characteristics and key findings of the 81 maternal and child published cohort studies conducted in the GCC countries, stratified by country.

**Additional file 4: Figure S1**. Modified Fig. 2 showing the pooled adjusted estimates on the association between maternal obesity and macrosomia after excluding two adjusted odds ratio estimates (1.53 and 9.18) that are converted to relative risk in Fig. 2 (1.19 and 1.12 respectively) reported by Wahabi HA et al. 2013 [49]. Note: Square indicates to the study-specific effect estimate. Size of the square is proportional to the precision (weight) of the study-specific effect estimate in the pooled estimate. Bars indicate the width of the 95% confidence interval (CI). The diamond centered on the summary effect estimate, and the width indicates the corresponding 95% CI of the pooled estimate.

**Additional file 5: Figure S2.** Modified Fig. 3 showing pooled adjusted estimates on the association between maternal obesity and CS delivery after excluding one estimate (4.80, 95% CI: 1.50–6.40) that is converted to relative risk in Fig. 3 (1.16) reported by Hassib YA., 2017 [47]. Note: Square indicates to the study-specific effect estimate. Size of the square is proportional to the precision (weight) of the study-specific effect estimate in the pooled estimate. Bars indicate the width of the 95% confidence interval (CI). The diamond centered on the summary effect estimate, and the width indicates the corresponding 95% CI of the pooled estimate.

**Additional file 6: Figure S3.** Summary of risk of bias (ROB) assessment of the 81 cohort studies using the NIH quality assessment tool for the cohort and cross-sectional studies.

**Additional file 7: Table S3.** Risk of bias (ROB) assessment of the 81 cohort studies using the NIH quality assessment tool for the cohort studies.

## Data Availability

The datasets used and/or analyzed during the current study and its additional information files are available from the corresponding author on reasonable request.

## Abbreviations

aOR: Adjusted odds ratio
aRRs: Adjusted relative risk
BMI: Body mass index
CI: Confidence interval
CVD: Cardiovascular diseases
DM: Diabetes mellitus
DNBC: Danish National Birth Cohort
GCC: Gulf Cooperation Council
GDM: Gestational diabetes mellitus
*I*^*2*^: I-squared
MoBa: Norwegian Mother and Child Cohort Study
NICU: Neonatal intensive care unit
NIH: National Heart, Lung, and Blood Institute
OR: Odds ratio
PECO: Participants, exposure, comparator, and outcome
PRISMA: Preferred Reporting Items for Systematic Review and Meta-Analysis
ROB: Risk of bias
RRs: Relative risk
SLE: Systemic lupus erythematosus
T1DM: Type 1 diabetes mellitus
T2DM: Type 2 diabetes mellitus
UAE: United Arab Emirates
WHO: World Health Organization
