# Supplementary material for: Maternal and birth cohort studies in the Gulf Cooperation Council countries: a systematic review and meta-analysis
Source: Syst Rev. 2020 Jan 16;9:14. doi: 10.1186/s13643-020-1277-0 (PMC6964097; doi:10.1186/s13643-020-1277-0)
Supplement: Supplementary file 1 — Additional file 1: Table S1. Preferred Reporting Items for Systematic Reviews and Meta-analyses (PRISMA) 2009 checklist [30]. [file 13643_2020_1277_MOESM1_ESM.docx]

PRISMA checklist. **Maternal and birth cohort studies in the Gulf Cooperation Council countries: a systematic review and meta-analysis**

| **Section/topic** | **#** | **Checklist item** | **Reported in main text on** |
| --- | --- | --- | --- |
| **TITLE** | | |  |
| Title | 1 | Identify the report as a systematic review, meta-analysis, or both.  The title page identifies this report as a systematic review and meta-analysis | p. 1 |
| **ABSTRACT** | | |  |
| Structured summary | 2 | Provide a structured summary including, as applicable: background; objectives; data sources; study eligibility criteria, participants, and interventions; study appraisal and synthesis methods; results; limitations; conclusions and implications of key findings; systematic review registration number.  The manuscript provides a structured summary as per the Obstetrics and Gynecology Journal’s guidelines. | p. 2 |
| **INTRODUCTION** | | |  |
| Rationale | 3 | Describe the rationale for the review in the context of what is already known. | p. 3-4 |
| Objectives | 4 | Provide an explicit statement of questions being addressed with reference to participants, interventions, comparisons, outcomes, and study design (PICOS).  In the third paragraph in the “Introduction” section, we clarified the objective of conducting this systematic review and meta-analysis. | p. 4 |
| **METHODS** | | |  |
| Protocol and registration | 5 | Indicate if a review protocol exists, if and where it can be accessed (e.g., Web address), and, if available, provide registration information including registration number.  A protocol for this review has been previously published as cited here:  Al-Rifai RH, Ali N, Barigye ET, et al. Maternal and birth cohort studies in the Gulf Cooperation Council countries: protocol for a systematic review and narrative evaluation. *BMJ Open* 2018; **8**(1): e019843. | P. 5 |
| Eligibility criteria | 6 | Specify study characteristics (e.g., PICOS, length of follow-up) and report characteristics (e.g., years considered, language, publication status) used as criteria for eligibility, giving rationale.  We specified the exact inclusion and exclusion criteria of studies to be included in this systematic review in the “Methods” section, under the subtitle “Search strategy and selection criteria”. | p. 6-8 |
| Information sources | 7 | Describe all information sources (e.g., databases with dates of coverage, contact with study authors to identify additional studies) in the search and date last searched.  We described that the information sources included the Medline (from 1945 to December 22, 2015) and EMBASE (from 1980 to December 22, 2015) databases in the “Methods” section, under the subtitle “Search strategy and selection criteria”. | p. 5-6 |
| Search | 8 | Present full electronic search strategy for at least one database, including any limits used, such that it could be repeated.  We summarised the full electronic search strategy for the Medline and EMBASE databases in “Box S1 in the appendix” and detailed it in the “Methods” section, under the subtitle “Data extraction and quality assessment”. | p. 4 & 5  S1 Box |
| Study selection | 9 | State the process for selecting studies (i.e., screening, eligibility, included in systematic review, and, if applicable, included in the meta-analysis).  We summarised the process for selecting studies in “Figure 1” and detailed it in “Methods” section, under the subtitle “Data extraction and quality assessment”. | p. 6  Figure 1 |
| Data collection process | 10 | Describe method of data extraction from reports (e.g., piloted forms, independently, in duplicate) and any processes for obtaining and confirming data from investigators.  We described the method of data extraction in the “Methods” section, under the subtitle “Data extraction and quality assessment”. | p. 6-7 |
| Data items | 11 | List and define all variables for which data were sought (e.g., PICOS, funding sources) and any assumptions and simplifications made.  In priori, we defined all variables to be extracted. We detailed that in the “Methods” section, under the subtitle “Data extraction and quality assessment”. | p. 6-7 |
| Risk of bias in individual studies | 12 | Describe methods used for assessing risk of bias of individual studies (including specification of whether this was done at the study or outcome level), and how this information is to be used in any data synthesis.  We evaluated the methodological quality and risk of bias (ROB) aspects for each cohort study using an established quality assessment tool for cohort studies. The NIH tool. | p. 9 |
| Summary measures | 13 | State the principal summary measures (e.g., risk ratio, difference in means).  We stated that the principle summary measure was the strength of association between TB and DM measured in the form of pooled adjusted odds ratios, hazard ratios, relative risks, or rate ratios. This was done in the “Methods” section, under the subtitle “Statistical analysis”. | p. 6 & 7 |
| Synthesis of results | 14 | Describe the methods of handling data and combining results of studies, if done, including measures of consistency (e.g., I^2^) for each meta-analysis. | p. 15 |
| Risk of bias across studies | 15 | Specify any assessment of risk of bias that may affect the cumulative evidence (e.g., publication bias, selective reporting within studies). | p. 9 |
| Additional analyses | 16 | Describe methods of additional analyses (e.g., sensitivity or subgroup analyses, meta-regression), if done, indicating which were pre-specified. | p. 10 |
| **RESULTS** | | |  |
| Study selection | 17 | Give numbers of studies screened, assessed for eligibility, and included in the review, with reasons for exclusions at each stage, ideally with a flow diagram.  We provided a flow diagram showing the number of studies screened, assessed for eligibility, and included in the review, with reasons for exclusions at each stage. Flow diagram is presented in “Figure 1”. | P. 10, and Figure 1 |
| Study characteristics | 18 | For each study, present characteristics for which data were extracted (e.g., study size, PICOS, follow-up period) and provide the citations.  We described the characteristics of the included studies in “Tables 1 and 2” and summarised those characteristics under the “Results” section. | p. 10-11 |
| Risk of bias within studies | 19 | Present data on risk of bias of each study and, if available, any outcome level assessment (see item 12).    We presented the data on risk of bias in “Fig S1, appendix 4 & S3 Table, appendix 5” with narrative information under the “Results” section. | P. 16 |
| Results of individual studies | 20 | For all outcomes considered (benefits or harms), present, for each study: (a) simple summary data for each intervention group (b) effect estimates and confidence intervals, ideally with a forest plot.  We presented simple summary estimate according to study design depicted in forest plots | Figure 2. Figure 3, Appendix 6_Fig S2, and Appendix 7_Fig S3 |
| Synthesis of results | 21 | Present results of each meta-analysis done, including confidence intervals and measures of consistency. | Figure 2. Figure 3, Appendix 6_Fig S2, and Appendix 7_Fig S3 |
| Risk of bias across studies | 22 | Present results of any assessment of risk of bias across studies (see Item 15). | Appendix 4_Fig S1 ROB, Appendix 5_S3 Table |
| Additional analysis | 23 | Give results of additional analyses, if done (e.g., sensitivity or subgroup analyses, meta-regression [see Item 16]). | Not applicable |
| **DISCUSSION** | | |  |
| Summary of evidence | 24 | Summarize the main findings including the strength of evidence for each main outcome; consider their relevance to key groups (e.g., healthcare providers, users, and policy makers).  The first paragraph in the “Discussion” section summarises the main findings of this systematic review. | p. 16 |
| Limitations | 25 | Discuss limitations at study and outcome level (e.g., risk of bias), and at review-level (e.g., incomplete retrieval of identified research, reporting bias).  We discussed the overall limitations of this systematic review in the second paragraph in the “Discussion” section. | p. 20-21 |
| Conclusions | 26 | Provide a general interpretation of the results in the context of other evidence, and implications for future research. | p. 21-22 |
| **FUNDING** | | |  |
| Funding | 27 | Describe sources of funding for the systematic review and other support (e.g., supply of data); role of funders for the systematic review.  This systematic review was supported by Zayed Center for Health Sciences (Grant Number 31R076) and the [United Arab Emirates University Program for Advanced Research (UPAR Grant Number 31M364)](https://www.uaeu.ac.ae/en/dvcrgs/research/rspo/opportunities.shtml#d9e87-4). No specific fund was received from the National Institutes of Health, Wellcome Trust, or Howard Hughes Medical Institute. | P. 26 |
