# Supplementary material for: Maternal and birth cohort studies in the Gulf Cooperation Council countries: a systematic review and meta-analysis
Source: Syst Rev. 2020 Jan 16;9:14. doi: 10.1186/s13643-020-1277-0 (PMC6964097; doi:10.1186/s13643-020-1277-0)
Supplement: Supplementary file 2 — Additional file 2: Box S1. Data sources and search criteria for systematically reviewing literature reporting on maternal and birth cohort studies in the GCC Countries. [file 13643_2020_1277_MOESM2_ESM.docx]

**S1 Box.** Databases search protocol and number of hits

| **Database** | **Search string** | **Search date** | **Applied filters** | **Search hits** |
| --- | --- | --- | --- | --- |
| **Medline via PubMed** | "cohort studies"[MeSH Terms] AND (((((("pregnancy"[MeSH Terms] OR "pregnancy"[All Fields]) OR ("pregnancy complications"[MeSH Terms] OR ("pregnancy"[All Fields] AND "complications"[All Fields]) OR "pregnancy complications"[All Fields])) OR ("maternal exposure"[MeSH Terms] OR ("maternal"[All Fields] AND "exposure"[All Fields]) OR "maternal exposure"[All Fields])) OR ("infant"[MeSH Terms] OR "infant"[All Fields])) OR ("child"[MeSH Terms] OR "child"[All Fields])) OR ("foetus"[All Fields] OR "fetus"[MeSH Terms] OR "fetus"[All Fields])) AND ((((("bahrain"[MeSH Terms] OR "kuwait"[MeSH Terms]) OR "oman"[MeSH Terms]) OR "qatar"[MeSH Terms]) OR "saudi arabia"[MeSH Terms]) OR "united arab emirates"[MeSH Terms]) | up to 30 June 2019 | no language restrictions, humans | 1,754 |
| **Embase-Ovid** | ('cohort':ab,ti AND pregnan*:ab,ti AND complication*:ab,ti OR maternal:ab,ti) AND (exposure*:ab,ti OR infant*:ab,ti OR child*:ab,ti OR fetus*:ab,ti) AND ('Bahrain' OR 'Kuwait' OR 'Oman' OR 'Qatar' OR 'Saudi Arabia' OR 'United Arab Emirates') | up to 30 June 2019 | no language restrictions, humans | 1,014 |
| **Scopus** | ((TITLE (cohort)  OR ABS (cohort))) AND (((TITLE (Bahrain OR Kuwait OR Oman OR Qatar OR "Saudi Arabia" OR “United Arab Emirates”) OR ABS (Bahrain OR Kuwait OR Oman OR Qatar OR "Saudi Arabia” OR "United Arab Emirates"))) AND ((TITLE (pregnancy OR "pregnancy complications" OR "maternal exposure” OR infant OR child OR fetus) OR ABS (pregnancy OR " pregnancy  complications” OR "maternal exposure" OR infant OR child OR fetus)))) | up to 30 June 2019 | no language restrictions, humans | 237 |
| **Web of Science** | (TI=cohort AND TI=(Pregnan* OR Pregnan* Complication* OR Mater* Exposur* OR Infant* OR Child* OR Fetus*) AND CU=("Bahrain" OR "Kuwait" OR "Oman" OR "Qatar" OR "Saudi Arabia" OR "United Arab Emirates")) | up to 30 June 2019 | no language restrictions, humans | 358 |
| **The Cochrane Library** | (Cohort AND (Pregnancy OR Pregnancy Complications OR Maternal Exposure OR Infant OR Child OR Fetus) AND ('Bahrain' OR 'Kuwait' OR 'Oman' OR 'Qatar' OR 'Saudi Arabia' OR 'United Arab Emirates')) | up to 30 June 2019 | no language restrictions, humans | 139 |
|  |  | **Total hits= 3,502**  **Removed duplicates: 509**  **Screened citations = 2,993** | | |
