# Supplementary material for: Maternal and birth cohort studies in the Gulf Cooperation Council countries: a systematic review and meta-analysis
Source: Syst Rev. 2020 Jan 16;9:14. doi: 10.1186/s13643-020-1277-0 (PMC6964097; doi:10.1186/s13643-020-1277-0)
Supplement: Supplementary file 3 — Additional file 3: Table S2. Summary characteristics and key findings of the 81 maternal and child published cohort studies conducted in the GCC countries, stratified by country. [file 13643_2020_1277_MOESM3_ESM.docx]

**S2 Table**: Studied populations, measured exposures/outcomes, and summary of key findings of the 81 maternal and child published cohort studies conducted in the GCC countries, stratified by country

| **Author, year** | **Studied population** | | **Measured exposure(s)** | | **Measured outcome(s)** | **Summary of key findings** | |
| --- | --- | --- | --- | --- | --- | --- | --- |
| **Bahrain** | |  | |  | | |  |
| Al Mahroos, S. et al., 2005 [22] | Non-diabetic pregnant women | | GDM ascertained after following a carbohydrate diet for three days | | MS, post-gravid insulin resistance, congenital anomaly, and still birth | Insulin resistance the possible cause of pathophysiological mechanism underlying the development of GDM continues in the post gravid state. | |
| **Kuwait** |  | |  | |  |  | |
| Pampaka, D. et al., 2019 [42] | Women with singleton pregnancies | | Antenatal depression and sociodemographic characteristics | | Postnatal depression | Antenatal depressive symptoms and other psychosocial characteristics predicted postnatal depressive symptoms | |
| Al Seaidan, M. et al., 2016 [94] | Pregnant mothers | | Nationality, age, parity, BMI, and smoking | | Still birth, preterm birth, small/ large for gestational age, and MS | Pre-pregnancy overweight and obese mothers were at a higher risk of at least one unfavorable birth outcome | |
| Scott, JA. et al., 2015 [95] | Mothers gave birth to live and healthy singleton babies at gestational age of ≥36 weeks | | Mother’s place of birth and feeding methods at 6 weeks | | Complementary feeding before 17 weeks of age | Women born in other Arab countries than Kuwait and who exclusively breast feed were less likely to introduce complementary foods to their babies before 17 weeks of age | |
| Dashti, M. et al., 2014 [24] | Postnatal mothers within 72 hours of delivery who gave birth to live and healthy singleton babies at gestational age of ≥36 weeks. | | Mother’s place of birth other several sociodemographics | | Breastfeeding | Maternal education, higher parity, demand feeding the infant in hospital, and father’s and grandmother’s preference for feeding increased breastfeeding duration while introduction to pacifier and return to work reduced breastfeeding duration. | |
| Al-Essa, M. et al., 2000 [21] | Newborns babies with a birth weight of >1,500 gram and gestational age <34 weeks | | Oxygen therapy, surfactant therapy, and presence of hypotension at birth | | Retinopathy of prematurity (ROP) | Oxygen therapy seemed to predispose the development of ROP. | |
| Al-Essa, M. et al., 2000 [20] | Preterm babies with a birth weight <1,501 grams (mean birth weight of 1,145.64 grams) | | Different neonatal and maternal exposure factors | | Severe (threshold stage) ROP | Threshold stage of retinopathy was associated with low birth weight, exposure to oxygen therapy, and septicemia. | |
| Al-Essa, M. et al., 1999 [96] | Preterm babies of <2,000 gram birth weight and/or <36 weeks gestational age | | Mean birth weight, oxygen therapy, presence of patient ductus arteriosus, presence of interventricular hemorrhage, blood transfusion, surfactant therapy, and phototherapy | | ROP | Low birth weight and oxygen therapy were the main risk factors for ROP in the study sample. | |
| Makhseed, M. et al., 1994 1994 [48] | Delivering mothers | | Previous CS, previous uterine scarring, and placenta previa | | Placenta previa, placenta accreta, postpartum hemorrhage, peri-partum hysterectomy, CS, and retained placenta | Women who underwent CS experienced an increase in placenta previa and accreta which in turn caused massive obstetric hemorrhage. Despite the increasing trend of previa and CS in Kuwait during the study period, the incidence of accreta did not increase. | |
| **Qatar** |  | |  | |  |  | |
| Bashir, M. et al., 2019 [43] | Pregnant women with/without T2DM | | T2DM | | Several maternal and neonatal outcomes | T2DM in pregnancy was associated with poor pregnancy maternal and neonatal outcomes compared to non-diabetic pregnant women. | |
| Bashir, M. et al., 2019 [44] | Pregnant women with early GDM (E-GDM diagnosed before 24 weeks of gestation) and usual GDM (U-GDM diagnosed after 24 weeks of gestation) | | E-GDM vs U-GDM | | Several maternal and neonatal outcomes | Pregnant women with E-GDM were older, more likely to be obese, more likely to need treatment and more likely to need supplemental insulin therapy compared with patients with U-GDM. Early screening for GDM in high-risk patients as early diagnosis and intervention was associated with better maternal and neonatal outcomes. | |
| Al-Obaidly, S. et al., 2019 [45] | Obese and morbid obese pregnant women in their third trimester | | Obesity (BMI = 30–39.9 kg/m^2^) and morbid obesity (BMI ≥40 kg/m^2^) | | Primary and repeated C-section, and birth weight | Increased rate of delivery by repeated CS deliveries. | |
| Bashir, M. et al., 2018 [97] | Pregnant women excluding women with known pre-conception diabetes. | | T2DM and GDM vs non-DM | | Several maternal and neonatal outcomes | Maternal and neonatal complications were higher among the diabetic (T2DM and GDM) compared to non-diabetic pregnant women in Qatar. | |
| Kunjachen Maducolil, M. et al., 2018 [98] | Stillbirths born on or after 24 weeks of gestation with no signs of life that occurred during the year 2015 | | Maternal characteristics | | Stillbirth | Proportion of stillbirths was higher in hypertensive, diabetic, and obese mothers . | |
| Bener, A. et al., 2013 [99] | Pregnant women in their third trimester | | Maternal age | | Anemia, GDM, gestational hypertension, ante partum hemorrhage, abruption placenta, placenta previa, pre-term birth, pre mature rupture of membranes, pre-eclampsia, APGAR score <1, Apgar score <5, and congenital anomaly | Older pregnant women >35 years were at a higher risk of different maternal complications and there was an increased risk of health issues amongsttheir neonates.  Note: the reported conclusions are inconsistent with the data presented in Table 3 and in the text in the original study. Column headings in Table 3 of the original study should be swapped. | |
| Bener, A. et al., 2011 [100] | Pregnant mothers | | Sociodemographic and obstetric characteristics | | GDM | Advanced maternal age, low monthly income, family history of diabetes, and obesity were the main risk factors for GDM. GDM also increased the risk of pregnancy complications such as pre-eclampsia and hemorrhage. | |
|  |  |  |  |  | Pre-eclampsia and hemorrhage |  |  |
| **Oman** |  | |  | |  |  | |
| Abdwani, R. et al., 2018 [108] | Neonates born to mothers with and without SLE | | SLE | | Several neonatal (e.g. birth weight and gestational age) and obstetrical (GDM, pre-eclampsia, oligohydramnios, and polyhydramnios) outcomes | Pregnancies in women with SLE were associated with higher neonatal and maternal complications. | |
| Zutshi, A. 2018 [109] | Pregnant obese and normal weight Omani women with available BMI data at < 12 gestational weeks | | Obesity | | Maternal and peri-natal (e.g. GDM and pre-eclampsia) and neonatal outcomes (e.g. congenital anomalies and birth weight) | Obese pregnant Omani women were at significantly increased risk of various maternal antenatal complications, intrapartum and postpartum events and neonatal complications. | |
| Al-Hakmani, FM. et al., 2016 [110] | Pregnant Omani women who presented to the primary health care center during their first trimester, between March 2011 and April 2012 | | BMI | | Maternal (mode and type of birth delivery, miscarriage, and GDM) and fetal (MS) outcomes | Maternal obesity in pregnant Omani women was associated with an increased risk of CS (especially elective cesarean), gestational hypertension, MS, and miscarriage. | |
| Abu-Heija, AT. et al., 2015 [111] | Pregnant Omani women aged between 15–49 years with GDM and Pre-GDM who delivered between January 2009 and December 2010 | | GDM (90.9%) and Pre-GDM (9.1%) | | Several obstetric (e.g. pre-eclampsia, preterm delivery at <37 weeks, CS, shoulder dystocia, polyhydramnios, induction of labour) and fetal (mean birth weight, birth weight and MS, IUGR, NICU admissions, neonatal hypoglycaemia) outcomes | Women with PGDM had a higher risk of developing obstetric complications such as pre-eclampsia or experiencing preterm or C-section deliveries in comparison to those with GDM | |
| Al-Farsi, YM. et al., 2011 [112] | Pregnant mothers | | Education, low income, and parity | | Anemia in pregnancy | Women with higher parity were at a higher risk of anemia during pregnancy. | |
| Barakat, MN. et al., 2010 [113] | Women with GDM, Pre-GDM (type 1 or type 2) and healthy women who gave birth between 1 January to 31 December 2004 | | GDM and pre-GDM vs. non-diabetic | | Maternal (type of delivery, condition of perineum) and fetal (infant maturity, birth weight, Apgar score at 1 minute, congenital anomalies, admission to special care baby unit) | Maternal GDM and pre-GDM were associated with several unfavorable maternal and fetal outcomes compared to healthy pregnant women. | |
| **Saudi Arabia** |  | |  | |  |  | |
| Al-Hathlol, K. 2018 [60] | Very low birth weight (VLBW) preterm infants admitted to the NICU with a birth weight of ≤1,500 g and gestational age of ≤ 32weeks | | In Vitro Fertilization (IVF) vs. spontaneous conception | | Neonatal and maternal outcomes | Among VLBW preterm infants, IVF had no detectable relationship with neonatal outcomes and duration of NICU stay. | |
| Mahzari, MM. et al., 2018 [61] | Women with a history of GDM who underwent postpartum diabetes screening | | History of GDM | | Postpartum T2DM | Older, multigravida, and multiparous women with history of GDM had a higher risk of developing T2DM | |
| Wahabi, HA. et al., 2017 [62] | Diabetic (pre-pregnancy type 1 or type 2 DM, or GDM) and non-diabetic Saudi pregnant mothers, with singleton pregnancies and gestational age of ≥24 weeks | | Pre-pregnancy T1DM or T2DM or GDM | | CS, induction of labor, MS, preterm delivery <37 weeks, still birth, shoulder dystocia, APGAR score <7 at 5 min, and admission to NICU | Saudi women with pre-pregnancy diabetes or GDM were at a higher risk of different maternal and neonatal morbidities and mortalities. Babies of pre-GDM mothers were more likely to be stillborn, admitted to NICU, or macrosomic. | |
| Almakoshi, A. et al., 2015 [70] | Two-year old Saudi children | | Maternal history of eczema, change in fetal abdominal circumference between second and third trimester, and increasing head circumference and abdominal circumference ratio between second and third trimester | | Eczema at 2 years | Between the second and third trimester, there was sub-optimal growth in the abdominal circumference and those with the lowest growth were at higher risk of eczema. | |
| Wahabi, HA. et al., 2014 [72] | Pregnant mothers with singleton pregnancy, gestational age of ≥37 weeks at the time of delivery, were free of type 1 or type 2 DM prior to the index pregnancy | | BMI, GDM | | Fetal MS (≥4 kg), birth weight, and CS | Maternal obesity and GDM independently and combined increased the risk of several adverse pregnancy outcomes including MS and CS. | |
| Wahabi, HA. et al., 2013 [75] | Postnatal women who gave birth to singleton baby at ≥37 weeks of gestation who did not smoke during the index pregnancy and who were or were not exposed to second-hand smoke | | BMI and exposure to second-hand smoke | | Newborn’s anthropometric measurements (birth weight, length, and head circumference) | Second-hand smoke and maternal obesity independently and combined increased the risk of neonatal complications including reduced anthropometric measures and a low or high birth weight, depending on the exposure. | |
| Wahabi, HA. et al., 2013 [76] | Singleton babies of Saudi mothers | | Age, parity and education | | Exposure to second-hand smoking | A third of pregnant women in Saudi Arabia were exposed to second-hand smoke which was associated with a lower birth weight and length. | |
|  |  |  | Maternal exposure during pregnancy to second hand smoke | | Birth weight and length of baby |  |  |
| Wahabi, HA. et al., 2013 [77] | Delivered mothers with GDM and non-diabetic | | GDM | | Maternal outcomes (emergency CS or CS delivery) and neonatal outcomes (APGAR score < 7 at 5 min, birth weight, MS, IUFD, and preterm delivery < 37 weeks | GDM was associated with an increased risk of CS and MS. | |
| Wahabi, HA. et al., 2012 [26] | Type 1 or type 2 and non-diabetic pregnant mothers with singleton pregnancies and gestational age of 24 weeks or more at the time of delivery | | Pre-pregnancy T1DM or T2DM | | Emergency CS, elective CS, birth weight, APGAR score at 5 min <7, MS, intrauterine fetal death, mean gestational age at delivery in weeks, and delivery <37 weeks | Pre-existing DM was associated with an increased risk of adverse maternal and perinatal outcomes including CS, MS, and preterm delivery . | |
| Al-Sunaidi MI and Al-Shabrani MS, 2011 [81] | Women pregnant with triplets confirmed by obstetric ultrasound scan | | Triplet pregnancies | | Maternal and neonatal complications | Good maternal and neonatal outcomes in triplet pregnancies. | |
| Al Arfaj AS and Khalil N, 2010 [27] | Pregnant mothers with systemic lupus erythromatosus (SLE) | | SLE features: Lupus nephritis, antiphospholipid (aPL), SLE flares in pregnancy, Anti-Ro antibodies, Hypertension, Anti-La antibodies, Raynaud’s, Taking prednisolone | | Pregnancy loss, pre-term birth, and intrauterine growth retardation | Women who had SLE and the risk factors mentioned in exposures had higher rates of adverse pregnancy outcomes such as pregnancy loss and preterm birth. | |
| Al-Mulhim, A. et al., 2003 [25] | Pregnant mothers | | Pre-eclampsia | | Maternal complications: placental abruption, oligonuria, renal failure, encephalopathy, HELLP syndrome, maternal death, transient blindness, and induced pregnancies and CS.  Neonatal outcomes: stillbirth and neonatal death. | Nulliparous women and women at the extreme ends of their reproductive years had a higher burden of pre-eclampsia. Positive association between severity of pre-eclampsia and maternal and neonatal complications. | |
| Al-Ajlan, A. et al., 2018 [59] | Pregnant Saudi women at high risk of GDM | | Maternal serum vitamin D status in first trimester | | GDM in second trimester | Saudi pregnant women with vitamin D deficiency were at a higher risk of developing GDM when  compared to their non-GDM counterparts. | |
| Alhainiah, MH. et al., 2018 [49] | Gradmultipara and Primigravida pregnant women. Gave birth between January and December 2016 | | Maternal parity (gradmultipara vs primigravida) | | Obstetrical complications) and neonatal (NICU, intrauterine fetal death, and neonatal death) | Maternal and neonatal adverse health outcomes were more common in grandmultipara compared to primigravida mothers. | |
| Al-Mouqdad, MM. et al., 2018 [50] | Premature newborns of gestational age ≤32 and birth weight of ≤1500 g along with suspected neonatal sepsis | | Duration of antibiotic administration (≤5 days vs. >5 days) | | Several neonatal outcomes (positive blood culture, bacterial resistance, Candidiasis, Necrotizing enterocolitis, and mortality) | Prolonged administration of empiric antibiotics to infants with very low birth weight along with sterile cultures was associated with the adverse outcomes late-onset sepsis and necrotizing enterocolitis. | |
| Hijazi, A. et al., 2018 [51] | Women who gave birth and who booked/not booked for antenatal care services | | Visiting antenatal care services | | Several ante-partum, intra-partum and post-partum complications | Antepartum and congenital anomalies were higher in booked mothers. No difference was found in antepartum, fetal and neonatal complications with four or less or more than four ANC visits. | |
| Baradwan, S. et al., 2018 [52] | Women presented with a history of infertility or recurrent pregnancy loss (Asherman’s syndrome), and treated by hysteroscopic adhesiolysis. | | Patients with endometrial thickness ≤5 mm | | Endometrial thick­ness and pregnancy | Adequate endometrial thickness is required for pregnancy to occur, and the preg­nancy rates were observed to be higher when the endome­trium was at least 5 mm in thickness among patients with Asherman’s syndrome. | |
|  |  |  | Patients with endometrial thickness >5 mm | |  |  |  |
| Al-Shaikh, GK. et al., 2017 [63] | Women who had singleton births excluding women with multiple gestations and several illnesses that might increase the risk of adverse pregnancy outcomes | | Parity (primipara, multipara, and grand multipara) | | Medical records | Grand multiparous Saudi females had similar risks of maternal and neonatal complications compared to the other parity groups. | |
| Magliah, SF. et al., 2019 [46] | Pregnant women with singleton pregnancies with/without GDM, and not known to be diagnosed with type 1 DM or type 2 DM prior to pregnancy | | GDM | | Maternal (induction of labor, vaginal delivery, emergency and elective C-section, and instrumental delivery) and neonatal (gestational age, birth weight, MS, low birth weight (<2500 g), and Apgar score of <7 at 5 minutes) outcomes | Women with GDM had no significant difference in terms of adverse maternal and neonatal outcomes as compared to non-diabetic. | |
| Serehi, AA. et al., 2015 [68] | Women admitted for delivery from April 2011 to March 2013 | | GDM/T2DM vs. non-DM | | Maternal (gestational age, pre-eclampsia, mode and type of birth delivery ) and fetal (APGAR Score at 5 and 10 minutes, birth weight, IUGR, fetal distress, congenital malformation, dystocia, fetal death, admission to NICU, and polyhydramnios) outcomes | Maternal and neonatal outcomes of patients with T2DM were more severe com­pared to GDM and non-DM Saudi patients. | |
| Alsammani, MA. and Ahmed, SR. 2015 [69] | Multipara and grand multipara women gave birth 01 January , 2012 through 31 December , 2012 | | Grand multiparity | | Maternal (mode of birth delivery, induced hypertension, premature rupture membranes, Placental Abruption/ previa, Postpartum hemorrhage) and neonatal (MS, admission to NICU) outcomes | Grand multiparity poses adverse maternal and neonatal pregnancy outcomes. | |
| Al-Qahtani, MH. 2014 [73] | Diabetic pregnant women who gave birth at the King Fahd University Hospital between May 2008 and April 2012 | | Type 1 and type 2, and GDM | | Maternal (mode of birth delivery ) and fetal (Apgar Score at 5 and 10 minutes, preterm birth, MS, hypoglycemia, hypocalcemia, polycythemia, acute respiratory distress syndrome, tachypnea, death, and small/ appropriate for gestational age) outcomes | Gestational DM continues as a health care problem with risks for both the mother and their offspring. The most common consequences of GDM were asymptomatic hypomagnesimia and hypocalcemia. | |
| Al Rowaily, MA. et al., 2014 [74] | Women who delivered by CS between 01 June 2008 and 28 February 2011 | | Sociodemographic and maternal characteristics | | Primary indication for CS and several maternal and fetal outcomes of CS | Difficult labor and previous CS were the main indications for CS in Saudi Arabia. Blood transfusion and IUGR were the most frequent adverse fetal outcomes. | |
| Gasim, T. 2012 [80] | Pregnant women with GDM | | GDM vs. non-diabetic pregnant women | | Maternal (gestational, preterm delivery, CS, polyhydramnios, oligohydramnios, induction of labor) and neonatal (mean birth weight, MS, NICU admissions >24 hours, *respiratory distress syndrome,* hypoglycemia at birth, neonatal Jaundice, phototherapy received, Apgar scores <7 at 5 minutes, neonatal anomalies, and perinatal deaths) outcomes | Maternal and neonatal outcomes in pregnant women with GDM in this study showed a significantly raised incidence of hypertensive disorders, CS, LGA neonates, MS and NICU admissions for >24 hours compared with the non-diabetic mothers. | |
| Al-Khalifah, R. et al., 2012 [79] | Pregnant women with GDM and without GDM who gave birth between January till December 2007 | | GDM vs. non-diabetic pregnant women | | Maternal (abortion, type of birth delivery) and neonatal (birth weight, Birth length, birth head circumference, admission to NICU, Apgar scores at 1 and 5 minutes, hypoglycemia, Jaundice, respiratory complications, CNS and cardiovascular complications) outcomes | GDM remains a significant morbidity to newborns resulting in increased intensive care admission, prolongation of hospital stay, and higher rates of neonatal hypoglycemia. | |
| Mansouri, HA. And Ghazawi, AH. 2007 [85] | Pregnant women with high order multiple pregnancies | | Multiple pregnancies (triplets, quadruples, and quintuplets) | | Maternal (bleeding in early trimester, pre-eclampsia, GDM, anemia, preterm labor, premature rupture of membranes, postpartum hemorrhage) and neonatal (death, admission to NICU, anomalies, mortality age at delivery, fetal distress) outcomes | High order multiple pregnancy was associated with a higher rate of perinatal mortality, which was mainly due to a higher rate of premature labor that resulted in low and very low birth weight newborns who are prone to complications and deaths. | |
| Yamani Zamzami, TY., 2004 [87] | Grand multiparous women who completed 37-week gestation or more with one previous CS and gave birth between 01 January 1991 and 31 December 2001 | | Maternal (age, gestational age, parity, height and weight, hypertension, DM, previous vaginal birth attempt) | | Maternal (vaginal birth after CS delivery) and neonatal (Apgar score at 1/5 minutes, fetal weight, still birth, and uterine dehiscence) outcomes | Vaginal birth after CS in grand multiparous women was common practice, safe, and efficacious. Multiparity in association with vaginal deliveries was a good prognostic factor, and predicted successful vaginal birth after CS outcome. | |
| Abdalrahman Almarzouki, A. 2003 [88] | Women with singleton pregnancies gave birth between June 2008 and November 2008 | | Controlled GDM with risk factors vs. no GDM with same risk factors | | Maternal (e.g. CS, hypertension, pre-term delivery) and neonatal (e.g. birth weight, Apgar score at 5 minutes, and respiratory distress) outcomes | Unfavorable pregnancy outcome were higher among controlled GDM in comparison to non-GDM women who had the same risk factors. | |
| Al-Jama, FE. et al., 1998 [47] | Pregnant patients with a known diagnosis of idiopathic thrombocytopenic recruited between January 1987 and December 1997 | | Idiopathic thrombocytopenic (4 months to 10 years) before the index pregnancy | | Maternal (corticosteroid therapy, splenectomy, antiplatelet antibodies, death, and mode of birth delivery) and neonatal (stillbirth, neonatal cord blood platelet count and fetal or neonatal hemorrhage) outcomes | The rarity of poor neonatal outcomes in mothers with ITP did not justify obstetric intervention solely on the basis of their platelet counts. The management of patients should be individualized and carefully planned | |
| Archibong, EI. Et al., 2003 [89] | Pregnant women admitted during the period January 1997– December 2001 with complaints of absent fetal movements | | Maternal characteristics (ante- antenatal care visits, gravidity) | | Stillbirth defined as delivery of a dead fetus weighing 500 g or more | Antenatal fetal deaths in the Asir obstetrics population were largely preventable. Attention should be paid to health education with emphasis on antenatal care and the benefits of regular clinic attendance, folic acid supplementation in pregnancy, family planning, and genetic counselling. | |
| Shawky, S. and Milaat, W. 2000 [90] | Pregnant teenage women married before the age of 16 years (mean marital age of 16±0.3 years). Data retrieved from medical records between March and April 1997 | | Teenage marriage | | Abortion, fetal death, and infant mortality | Teenage marriage posed several maternal and neonatal health and mortality risks. | |
| El Mallah, KO. Et al., 1997 [92] | Women with GDM and pre-GDM and non-diabetic women who gave birth between January 1991 and April 1994 | | GDM and pre-GDM | | Mode of delivery and peripartum and perinatal complications (e.g. stillbirths, MS, perineal lacerations, pre-eclampsia) | The incidence of maternal, fetal, and neonatal complications in GDM was similar to pre-GDM patients and their offspring. | |
| Meshari, AA. et al., 1990 [41] | Pregnant women who their antenatal care at King Abdul Aziz teaching hospital in Riyadh from January 1984 to December 1986 | | Maternal (age, parity, height, Ponderal index, gestational weeks, diabetes, and hypertension) | | Maternal (mode of birth delivery, prolonged labour, postpartum hemorrhage) and eonatal (MS, shoulder dystocia, perinatal mortality, Apgar score, jaundice, and hypoglycemia) outcomes | Maternal obesity, grand multiparity, diabetes mellitus, and post-maturity were the major maternal risks. | |
| Eltawel, M. et al., 2018 [53] | Newborns (up to 7 days post-delivery) with neonatal thrombocyto­penia (platelet counts of fewer than 150,000/μL of blood) | | Fetomaternal and neonatal characteristics | | Onset of thrombocyto­penia, and neonatal recovery or death (duration of recovery) | Neonatal thrombocytopenia generally emerged in less than 72 hours. Lower gestational ages was associated with the rapid onset and negative neonatal thrombocyto­penia | |
| Ellaithy, M. et al., 2018 [54] | Women with a primary tubal ectopic pregnancy excluding women with non-tubal ectopic pregnancy and use of contraception | | Maternal and medical | | Recurrent ectopic pregnancy | Prior miscarriage, evacuation of retained products of conception, or pelvic inflammatory disease tripled the risk of ectopic pregnancy whereas consanguinity doubled this risk ectopic pregnancy. Primary ectopic pregnancy data is a valuable tool to predict the potential risk of recurrence in the future. | |
| Shalaby, MA. et al., 2018 [55] | Neonates who were admitted to NICU during 2015 (January to December) | | Neonatal characteristics (gestational age, peri-natal depression, exposure to nephrotoxic drugs, and sepsis) | | Acute Kidney Injury (AKI) | Neonates with evidence of AKI had lower birth weight, lower Gestational age, and were sicker at birth. Neonatal AKI was associated with increased mortality and length of hospital stay. Improving neonatal outcomes requires the identification of neonates at a high-risk of AKI | |
| Sobaih, BH. 2018 [56] | Very low birth weight infants (1000-1500 grams) | | Neonatal birth weight | | Cognitive function outcome, sepsis, blindness, deafness, and cerebral palsy | Cerebral palsy and being male, lower gestational age, and lower birth weight infant increased the risk of future cognitive impairment. | |
| Al-Nemri, A. M. et al., 2018 [57] | Neonates excluding those born at 23 weeks or less, who died within 3 hours of delivery, twins, and unbooked pregnant women | | Diabetes mellitus (gestational, type 1, and type 2) and HbA1c level in each of the three pregnancy trimesters | | Perinatal maternal and neonatal morbidity | Neonates of diabetic mothers developed a variety of neonatal events that largely correlated with poor metabolic control during pregnancy. Controlling blood glucose level in pregnant women would help in averting several neonatal and maternal outcomes. | |
| Al‑Qashar, F. et al., 2018 [58] | Singleton infants born complicated with intrauterine growth restriction with a birth weight <10^th^ percentile | | Symmetrical and asymmetrical intrauterine growth restriction | | Neurodevelopmental impairment | Intrauterine growth restriction was an independent variable for poor neurodevelopmental outcome. | |
| Fayed, AA. et al., 2017 [64] | Pregnant mothers recruited in RAHMA cohort study | | Maternal age | | Premature birth, CS, low birth weight, MS, GDM, pregnancy associated hypertension, NICU admission, induction of labor, and still birth | Older women experienced adverse pregnancy outcomes such as preterm delivery, GDM, and CS. However, younger women experienced issues such as preterm delivery. | |
| Haseeb, YA. 2017 [65] | Obese (BMI ≥29.9 kg/m^2^ ) and non-obese (BMI <29.9 kg/m^2^ ) Saudi pregnant females in theirs first trimester of pregnancy | | Obesity in mother | | Maternal (pre-eclampsia, antepartum hemorrhage, GDM, postpartum hemorrhage, CS) and fetal (MS, shoulder dystocia, birth asphyxia of severe degree, premature birth, NICU admission, wound complications, and thromboembolism | Obese pregnant women experienced a higher rate of morbidities such as GDM, CS, and thromboembolism, compared to non-obese pregnant women. | |
| Saad Waheeb and Kahlid Alshehri, 2016 [66] | Premature births (born ≤32 weeks gestational age and/or birth weight ≤1500 grams) with a mean gestational age of 26.7 weeks | | Patent ductus arteriosus (PDA), Intraventricular hemorrhage (IVH), hydrocephalus, and sepsis | | ROP or the retina was fully vascularized | PDA and IVH were risk factors for ROP. Laser therapy was an effective treatment for ROP. | |
| Alfadhli, EM. et al., 2015 [67] | Pregnant women treated in the antenatal service during October 2011 to June 2014 | | GDM at the first antenatal visit | | Several maternal and neonatal outcomes (e.g. abortion, stillbirth, neonatal death, birth weight, Apgar score at 5 min, neonatal) | Pregnant Saudi women with GDM were at a higher risk of several maternal and neonatal adverse health outcomes | |
| Lasheen, AE. et al., 2014 [71] | Newborns of diabetic mothers with either pre-GDM or GDM | | Diabetic pregnant mothers (pre-gestational or GDM) | | Length of stay in hospital, congenital malformation, congenital heart diseases, preterm low birth weight, traumatic injury, respiratory, sepsis, hypoglycemia, hyperbilirubinemia, and hypocalcemia | Diabetic mothers had a greater likelihood of experiencing birth complications including congenital malformations and injury compared to non-diabetic mothers. | |
|  |  |  | Non-diabetic pregnant mothers with no other medical disorders | |  |  |  |
| Hammouda, SA. et al., 2013 [78] | Native Saudi-Arabian pregnant mothers in their first trimester without systematic diseases that could cause congenital malformation | | Serum level of micronutrients (vitamin E, vitamin A, and total folic acid, and vitamin B12) and trace elements (selenium, zinc, magnesium, and homocysteine) | | Congenital malformation in offspring | Micronutrient deficiencies during pregnancy were associated with congenital malformations in the infant. These included selenium, zinc, magnesium, vitamins A, B12, and folic acid. | |
| El-Gilany A. and Hammad S, 2010 [82] | Pregnant mothers in their first month of pregnancy excluding women with any pre-pregnancy chronic medical conditions or with multiple pregnancies | | BMI during the first ANC visit | | Pregnancy-induced hypertension, GDM, ante-partum hemorrhage, pre-eclamptic toxemia, anemia, Urinary Tract Infections (UTIs), route of delivery, gestational age at birth, stillbirth, birth weight, 1-minute APGAR score, and NICU | Antenatal: obese and overweight mothers had an increased risk of pregnancy-induced hypertension, GDM, pre-eclamptic toxemia, urinary tract infection, and CS. | |
|  |  |  |  |  |  | Neonatal: neonates born to obese mothers had an increased risk of postdate pregnancy (>42 weeks), MS, low 1-minute Apgar score, and admission to NICU. Neonates born to underweight mothers had an increased risk of low birth weight. | |
| Al-Saleh, I. et al., 2009 [83] | Healthy Saudi pregnant women with a mean age of 28.5 years | | Low prenatal exposure to lead | | Early cognitive development of infants at different early life stages of 6, 12, 18, and 24 months | Prenatal lead exposure was found to be associated with cognitive development. | |
|  |  |  | High prenatal exposure to lead | |  |  |  |
| Al-Saleh, I. et al., 2008 [84] | Newborns of Saudi women with a mean age of 28.5 years at time of delivery | | Maternal age, parity, residence, and prenatal supplements  Cord blood levels | | Cord blood levels  Newborn head circumference | Maternal age and parity were positively correlated with cord blood lead levels. Prenatal supplement intake was negatively associated with cord blood lead levels which emphasis the role of supplements intake during pregnancy | |
| Al-Hakeem, MM. 2006 [86] | Pregnant women who gave birth between January 2000 and December 2001 and diagnosed with GDM who were either on dietary modification or insulin treatment | | GDM | | Maternal and neonatal outcomes | GDM was associated with several unfavorable maternal and neonatal outcomes. Proper measures could be taken to reduce complications during delivery and the neonatal period and thereby, minimize particularly NICU admission rates. | |
| Al-Dabbous, I. A. et al., 1996 [91] | Diabetic mothers (on insulin and not on insulin therapy during pregnancy over a 5-year (1988-1992) period | | Maternal DM | | Several neonatal (e.g. birth weight, preterm births, congenital anomalies) and maternal (polyhydraminos, premature rupture of membranes, pre-eclampsia, shoulder dystocia, and hyperthyroidism) outcomes | Poor maternal diabetic control resulted in high perinatal morbidity and mortality in the offspring of diabetic mothers. | |
| Alfonso, F. et al., 1990 [40] | Pregnant women who attended the department of Obstetrics and Gynecology at KKUH, between January 2000 and December 2001 | | GDM | | Maternal (type of birth and gestational age) and neonatal (admission to NICU, birth weight, preterm labour, and Apgar score at 1 and 5 minutes) outcomes | Pregnancies complicated by GDM without treatment are associated with a higher frequency of adverse maternal and fetal outcomes. | |
| Fayed, HM. et al., 1993 [93] | Multipara women who gave birth between June 1986 and May 1991 at the Security Forces Hospital, Riyadh. | | Multiparity (extreme ≥ 10 vs. 2-5) | | Maternal (prolonged gestation, placenta previa/abruption, postpartum hemorrhage, CS, perinatal mortality) and neonatal (Apgar score, weight, MS, anomalies, fetal presentation) | Extreme grand multiparity did not carry any added special obstetric or perinatal risk if the mother was from a high socioeconomic background and received a high standard of antenatal care. | |
| **UAE** |  | |  | |  |  | |
| Gardner, H. et al., 2015 [102] | Emirati mothers with a mean age of 28.7 years at birth and a mean age at marriage of 20.8 years | | Sociodemographic (education, age, education) | | Supplementary feeding at 3 months, and mother still breastfeeding at 15 months | Factors such as time to breastfeed, mother’s education level, employment status, and the early introduction to complementary foods were associated with early cessation of breastfeeding. | |
| Hamdan A. and Tamim H. 2011 [103] | Emirati and non-Emirati pregnant mothers | | Parity, religion, depression using Beck’s Depression Inventory-II, Beck Anxiety Inventory, and Life Events Inventory | | Depression based on Mini International Neuropsychiatric Interview (MINI) and Edinburgh Postnatal Depression Scale (EPDS) | Postpartum depression was associated with having depression during the second and third trimester, number of children, religion, and formula feeding. | |
| Al Tajir, GK. et al., [104] | Emirati and non-Emirati delivering mothers | | Education, nationality, pethidine exposure, parity, age, and length of hospital stay | | Exclusive breastfeeding at day 1, 1 month, and 6 months | Factors such as nationality and pethidine exposure were associated with breastfeeding exclusively. Education level was the factor most strongly associated with exclusive breastfeeding behavior at 6 months | |
| Sharief, NM. et al., [106] | Emirati and non-Emirati delivering mothers | | Education | | Continuation of breastfeeding at six months after delivery. | Proportion of mothers who continued breastfeeding at six months after delivery significantly differed according to the level of education. | |
|  |  | |  | |  |  | |
| Gardner, H. et al., 2018 [101] | Infants of Emirati women | | Maternal and medical | | Birth weight <2.5 kg | The health of infants born to the mothers in this UAE sample from Abu Dhabi showed marked improvement over previous studies. Although consanguinity among parents was high, no evidence for negative impacts on birth weight or prematurity was found. | |
| Fareh, OI. et al., 2005 [105] | Pregnant women with and without anaemia during pregnancy | | Anaemia during pregnancy defined as a haemoglobin level <11 g/dl irrespective of the period of gestation | | Maternal (post-partum haemorrhage, preterm delivery, required blood transfusion, and post-term pregnancy) and neonatal (fetal growth restriction, APGAR score at 1 and 5 minutes, and birth weight) outcomes | Anaemia during pregnancy was associated with increased likelihood of receiving blood transfusion in the postnatal period particularly if post-partum haemorrhage occurred. | |
| Al-Ali, FM. et al., 1997 [107] | Babies with an age range of 3–17 months | | Breastfeeding | | Diarrhea | Breastfeeding played an important role in reducing the impact of infantile diarrhea, regardless of improved sanitation and improved water supply. | |

APGAR: appearance, pulse, grimace, activity, respiration; APH: antepartum hemorrhage; aPL: antiphospholipid; BDI: The Beck Depression Inventory-II; BMI: body mass index; C-section: caesarean section; DM: diabetes mellitus; EPDS: edinburgh post-natal depression score; IUFD: intrauterine fetal death; LBW: low birth weight; MINI: mini international neuropsychiatric inventory; NICU: neonatal intensive care unit; GDM: gestational diabetes mellitus;G6PD: glucose-6-phosphate dehydrogenase; ROP: retinopathy of prematurity; SLE: systemic lupus erythematosus; PDA: patent ductus arteriosus
