# Supplementary figures and images for: Maternal and birth cohort studies in the Gulf Cooperation Council countries: a systematic review and meta-analysis
Source: Syst Rev. 2020 Jan 16;9:14. doi: 10.1186/s13643-020-1277-0 (PMC6964097; doi:10.1186/s13643-020-1277-0)

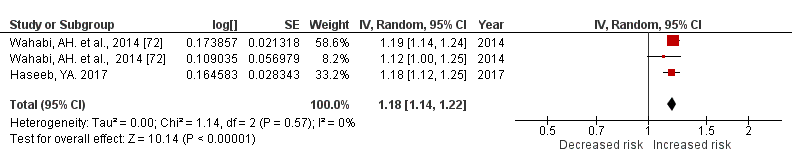


[65]

Supplement: Supplementary file 4 — Additional file 4: Figure S1. Modified Fig. 2 showing the pooled adjusted estimates on the association between maternal obesity and macrosomia after excluding two adjusted odds ratio estimates (1.53 and 9.18) that are converted to relative risk in Fig. 2 (1.19 and 1.12 respectively) reported by Wahabi HA et al. 2013 [49]. Note: Square indicates to the study-specific effect estimate. Size of the square is proportional to the precision (weight) of the study-specific effect estimate in the pooled estimate. Bars indicate the width of the 95% confidence interval (CI). The diamond centered on the summary effect estimate, and the width indicates the corresponding 95% CI of the pooled estimate. [file 13643_2020_1277_MOESM4_ESM.docx]

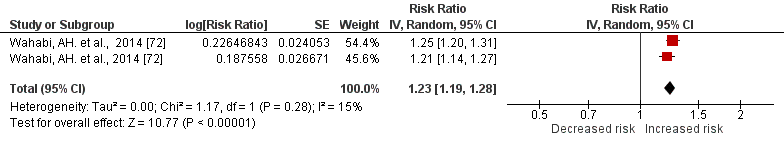

Supplement: Supplementary file 5 — Additional file 5: Figure S2. Modified Fig. 3 showing pooled adjusted estimates on the association between maternal obesity and CS delivery after excluding one estimate (4.80, 95% CI: 1.50–6.40) that is converted to relative risk in Fig. 3 (1.16) reported by Hassib YA., 2017 [47]. Note: Square indicates to the study-specific effect estimate. Size of the square is proportional to the precision (weight) of the study-specific effect estimate in the pooled estimate. Bars indicate the width of the 95% confidence interval (CI). The diamond centered on the summary effect estimate, and the width indicates the corresponding 95% CI of the pooled estimate. [file 13643_2020_1277_MOESM5_ESM.docx]
