# Supplementary material for: Maternal and birth cohort studies in the Gulf Cooperation Council countries: a systematic review and meta-analysis
Source: Syst Rev. 2020 Jan 16;9:14. doi: 10.1186/s13643-020-1277-0 (PMC6964097; doi:10.1186/s13643-020-1277-0)
Supplement: Supplementary file 7 — Additional file 7: Table S3. Risk of bias (ROB) assessment of the 81 cohort studies using the NIH quality assessment tool for the cohort studies. [file 13643_2020_1277_MOESM7_ESM.docx]

**S3 Table**: Summary ROB assessment of the 81 maternal and child published cohort studies conducted in the GCC countries

| **Author, year** | **Criteria** | | | | | | | | | | | | | | | | | | | | **Total criteria with potentially** | | | |  |
| --- | --- | --- | --- | --- | --- | --- | --- | --- | --- | --- | --- | --- | --- | --- | --- | --- | --- | --- | --- | --- | --- | --- | --- | --- | --- |
|  | Q 1 | Q 2 | Q 3 | Q 4 | Q 5 | Q 6 | Q 7 | Q 8 | | Q 9 | | Q 10 | | Q 11 | | Q 12 | Q 13 | | Q 14 | | Low  ROB | | High ROB | |  |
| ***Prospective cohort studies*** | | | |  |  |  |  |  | |  | |  | |  | |  |  | |  | |  | |  | |  |
| Ellaithy, M. et al., 2018 [54] | 1 | 1 | 1 | 1 | 1 | 1 | 2 | 1 | | 1 | | 0 | | 1 | | 2 | 1 | | 1 | | 11 | | 1 | |  |
| Al-Nemri, AM. et al., 2018 [57] | 1 | 1 | 1 | 1 | 0 | 1 | 1 | 1 | | 1 | | 3 | | 1 | | 2 | 1 | | 0 | | 10 | | 1 | |  |
| Sobaih, BH. 2018 [56] | 1 | 1 | 1 | 0 | 1 | 1 | 1 | 1 | | 1 | | 0 | | 1 | | 2 | 0 | | 0 | | 9 | | 3 | |  |
| Shalaby, MA. et al., 2018 [55] | 1 | 1 | 2 | 1 | 0 | 1 | 2 | 1 | | 1 | | 2 | | 1 | | 2 | 1 | | 1 | | 9 | | 1 | |  |
| Pampaka, D. et al., 2019 [42] | 1 | 1 | 1 | 1 | 0 | 1 | 2 | 1 | | 1 | | 0 | | 1 | | 2 | 0 | | 1 | | 8 | | 3 | |  |
| Eltawel, M. et al., 2018 [53] | 1 | 1 | 1 | 1 | 0 | 1 | 1 | 1 | | 1 | | 3 | | 1 | | 2 | 1 | | 1 | | 11 | | 1 | |  |
| Gardner, H. et al., 2018 [101] | 1 | 1 | 1 | 1 | 0 | 1 | 1 | 1 | | 1 | | 3 | | 1 | | 2 | 1 | | 1 | | 11 | | 1 | |  |
| Al‑Qashar, F. et al., 2018 [58] | 1 | 1 | 2 | 1 | 0 | 1 | 1 | 1 | | 1 | | 2 | | 1 | | 2 | 1 | | 0 | | 9 | | 2 | |  |
| Al Mahroos, S. et al., 2005 [22] | 1 | 1 | 1 | 1 | 0 | 1 | 1 | 0 | | 1 | | 3 | | 1 | | 2 | 0 | | 0 | | 8 | | 4 | |  |
| Al Seaidan, M. et al., 2016 [94] | 1 | 1 | 0 | 1 | 0 | 1 | 1 | 1 | | 1 | | 3 | | 1 | | 2 | 1 | | 0 | | 9 | | 3 | |  |
| Scott, JA. et al., 2015 [95] | 1 | 1 | 1 | 1 | 1 | 1 | 1 | 1 | | 1 | | 1 | | 1 | | 2 | 0 | | 1 | | 12 | | 1 | |  |
| Dashti, M. et al., 2014 [24] | 1 | 1 | 2 | 1 | 1 | 1 | 1 | 1 | | 1 | | 3 | | 1 | | 2 | 2 | | 1 | | 10 | | 0 | |  |
| Al-Essa, M. et al., 2000 [21] | 1 | 1 | 1 | 1 | 1 | 1 | 1 | 1 | | 1 | | 3 | | 1 | | 2 | 1 | | 1 | | 12 | | 0 | |  |
| Al-Essa, M. et al., 2000 [20] | 1 | 1 | 3 | 1 | 1 | 1 | 1 | 1 | | 1 | | 3 | | 1 | | 4 | 3 | | 1 | | 10 | | 0 | |  |
| Al-Essa, M. et al., 1999 [96] | 1 | 1 | 1 | 1 | 0 | 1 | 2 | 1 | | 1 | | 3 | | 1 | | 4 | 1 | | 0 | | 9 | | 2 | |  |
| Bener, A. et al., 2013 [99] | 1 | 1 | 1 | 1 | 1 | 0 | 2 | 1 | | 1 | | 3 | | 1 | | 4 | 1 | | 0 | | 9 | | 2 | |  |
| Bener, A. et al., 2011 [100] | 1 | 1 | 1 | 1 | 1 | 1 | 1 | 1 | | 1 | | 0 | | 1 | | 2 | 4 | | 1 | | 11 | | 1 | |  |
| Fayed, AA. et al., 2017 [64] | 1 | 1 | 3 | 1 | 1 | 1 | 1 | 1 | | 1 | | 3 | | 1 | | 4 | 3 | | 1 | | 10 | | 0 | |  |
| Haseeb, YA. 2017 [65] | 1 | 1 | 2 | 1 | 1 | 1 | 1 | 3 | | 1 | | 3 | | 0 | | 2 | 1 | | 1 | | 8 | | 1 | |  |
| Waheeb S and Kahlid Alshehri, 2016 [66] | 1 | 1 | 1 | 1 | 1 | 1 | 2 | 3 | | 1 | | 3 | | 1 | | 4 | 2 | | 1 | | 9 | | 0 | |  |
| Lasheen, AE. et al., 2014 [71] | 1 | 1 | 4 | 1 | 0 | 1 | 2 | 1 | | 1 | | 3 | | 1 | | 4 | 1 | | 0 | | 8 | | 2 | |  |
| Hammouda, SA. et al., 2013 [78] | 1 | 1 | 1 | 1 | 1 | 1 | 1 | 1 | | 1 | | 0 | | 1 | | 4 | 1 | | 0 | | 11 | | 2 | |  |
| El-Gilany A. and Hammad S, 2010 [82] | 1 | 1 | 1 | 1 | 0 | 1 | 1 | 1 | | 1 | | 3 | | 1 | | 4 | 1 | | 0 | | 10 | | 2 | |  |
| Al-Saleh, I. et al., 2009 [83] | 1 | 1 | 2 | 1 | 0 | 1 | 1 | 1 | | 1 | | 1 | | 1 | | 4 | 2 | | 1 | | 10 | | 1 | |  |
| Al-Saleh, I. et al., 2008 [84] | 1 | 1 | 2 | 1 | 0 | 1 | 1 | 0 | | 1 | | 0 | | 1 | | 2 | 4 | | 1 | | 8 | | 3 | |  |
| Gardner, H. et al., 2015 [102] | 1 | 1 | 1 | 1 | 0 | 1 | 1 | 1 | | 1 | | 3 | | 2 | | 4 | 0 | | 1 | | 9 | | 2 | |  |
| Hamdan A. and Tamim H. 2011 [103] | 1 | 1 | 1 | 1 | 0 | 1 | 2 | 1 | | 1 | | 1 | | 1 | | 2 | 1 | | 0 | | 10 | | 2 | |  |
| Al Tajir, GK. et al., 2006 [104] | 1 | 1 | 2 | 1 | 0 | 0 | 1 | 0 | | 0 | | 3 | | 1 | | 4 | 1 | | 1 | | 7 | | 4 | |  |
| Sharief, NM. et al., 2001 [106] | 1 | 1 | 1 | 1 | 0 | 1 | 1 | 1 | | 0 | | 3 | | 0 | | 4 | 1 | | 0 | | 8 | | 4 | |  |
| Alfadhli, EM. et al., 2015 [67] | 1 | 1 | 1 | 1 | 0 | 1 | 1 | 1 | | 1 | | 0 | | 1 | | 2 | 1 | | 0 | | 9 | | 3 | |  |
| Al-Jama, FE. et al., 1998 [47] | 1 | 1 | 1 | 1 | 0 | 1 | 1 | 2 | | 1 | | 3 | | 1 | | 2 | 1 | | 0 | | 9 | | 2 | |  |
| Alfonso, F. et al., 1990 [40] | 1 | 1 | 1 | 1 | 0 | 1 | 1 | 3 | | 1 | | 0 | | 1 | | 2 | 1 | | 0 | | 8 | | 3 | |  |
| Meshari, AA. et al., 1990 [41] | 1 | 1 | 1 | 1 | 0 | 1 | 1 | 1 | | 1 | | 0 | | 1 | | 2 | 1 | | 0 | | 9 | | 3 | |  |
| *Mean number of criteria* | *–* | *–* | *–* | *–* | *–* | *–* | *–* | *–* | | *–* | | *–* | | *–* | | *–* | *–* | | *–* | | *9.42* | | *1.8* | |  |
| ***Retrospective cohort studies*** | | | |  |  |  |  |  | |  | |  | |  | |  |  | |  | |  | |  | |  |
| Bashir, M. et al., 2019 [43] | 1 | 1 | 1 | 1 | 0 | 1 | 1 | 1 | | 1 | | 3 | | 1 | | 2 | 1 | | 1 | | 11 | | 1 | |  |
| Bashir, M. et al., 2019 [44] | 1 | 1 | 1 | 1 | 0 | 1 | 1 | 1 | | 1 | | 3 | | 1 | | 2 | 1 | | 1 | | 11 | | 1 | |  |
| Bashir, M. et al., 2018 [97] | 1 | 1 | 1 | 1 | 0 | 1 | 1 | 1 | | 1 | | 3 | | 1 | | 2 | 1 | | 0 | | 10 | | 2 | |  |
| Baradwan, S. et al., 2018 [52] | 1 | 1 | 1 | 1 | 0 | 1 | 1 | 1 | | 1 | | 3 | | 1 | | 2 | 1 | | 0 | | 10 | | 2 | |  |
| Al-Shaikh, GK. et al., 2017 [63] | 1 | 1 | 1 | 1 | 0 | 1 | 1 | 1 | | 1 | | 3 | | 1 | | 2 | 1 | | 1 | | 10 | | 1 | |  |
| Magliah, SF. et al., 2019 [46] | 1 | 1 | 2 | 1 | 1 | 1 | 1 | 1 | | 1 | | 0 | | 1 | | 2 | 1 | | 0 | | 10 | | 2 | |  |
| Al-Obaidly, S. et al., 2019 [45] | 1 | 1 | 2 | 1 | 0 | 1 | 1 | 1 | | 1 | | 3 | | 1 | | 2 | 3 | | 0 | | 8 | | 2 | |  |
| Abdwani, R. et al., 2018 [108] | 1 | 1 | 2 | 1 | 0 | 1 | 1 | 1 | | 1 | | 0 | | 1 | | 2 | 3 | | 0 | | 8 | | 3 | |  |
| Al-Ajlan, A. et al., 2018 [59] | 1 | 1 | 2 | 1 | 1 | 1 | 1 | 1 | | 1 | | 0 | | 1 | | 3 | 1 | | 1 | | 11 | | 1 | |  |
| Al-Hathlol, K. 2018 [60] | 1 | 1 | 2 | 1 | 0 | 1 | 1 | 1 | | 1 | | 3 | | 1 | | 2 | 1 | | 1 | | 11 | | 1 | |  |
| Zutshi, A. 2018 [109] | 1 | 1 | 2 | 1 | 0 | 1 | 1 | 1 | | 1 | | 0 | | 1 | | 2 | 1 | | 0 | | 9 | | 3 | |  |
| Hijazi, A. et al., 2018 [51] | 1 | 1 | 1 | 1 | 1 | 1 | 1 | 1 | | 1 | | 0 | | 1 | | 2 | 1 | | 1 | | 12 | | 1 | |  |
| Kunjachen Maducolil, M. et al., [98] | 1 | 1 | 1 | 1 | 0 | 1 | 1 | 1 | | 3 | | 1 | | 2 | | 1 | 1 | | 0 | | 10 | | 2 | |  |
| Mahzari, MM. et al., 2018 [61] | 1 | 1 | 1 | 1 | 0 | 1 | 2 | 1 | | 1 | | 3 | | 1 | | 2 | 1 | | 0 | | 8 | | 2 | |  |
| Al-Mouqdad, MM. et al., 2018 [50] | 1 | 1 | 1 | 1 | 0 | 1 | 2 | 1 | | 1 | | 3 | | 1 | | 2 | 1 | | 0 | | 8 | | 2 | |  |
| Serehi, AA. et al., 2015 [68] | 1 | 1 | 1 | 1 | 0 | 1 | 1 | 1 | | 2 | | 1 | | 2 | | 1 | 1 | | 0 | | 9 | | 2 | |  |
| Abu-Heija, AT. Et al., 2015 [111] | 1 | 1 | 1 | 1 | 0 | 1 | 1 | 1 | | 2 | | 1 | | 1 | | 2 | 1 | | 0 | | 10 | | 2 | |  |
| Archibong, EI. Et al., 2003 [89] | 1 | 1 | 1 | 1 | 0 | 1 | 1 | 1 | | 1 | | 0 | | 1 | | 2 | 1 | | 0 | | 10 | | 3 | |  |
| Fareh, OI. et al., 2005 [105] | 1 | 1 | 1 | 1 | 1 | 1 | 1 | 1 | | 1 | | 3 | | 1 | | 2 | 1 | | 0 | | 11 | | 1 | |  |
| Gasim, T. 2012 [80] | 1 | 1 | 1 | 1 | 0 | 1 | 1 | 1 | | 1 | | 3 | | 1 | | 2 | 1 | | 0 | | 11 | | 1 | |  |
| Al-Qahtani, MH. 2014 [73] | 1 | 1 | 1 | 1 | 0 | 1 | 1 | 3 | | 1 | | 3 | | 1 | | 2 | 1 | | 0 | | 8 | | 2 | |  |
| al-Dabbous, IA. et al., 1996 [91] | 1 | 1 | 1 | 1 | 0 | 1 | 1 | 3 | | 1 | | 3 | | 1 | | 2 | 1 | | 0 | | 8 | | 2 | |  |
| Al-Khalifah, R. et al., 2012 [79] | 1 | 1 | 1 | 1 | 0 | 1 | 1 | 3 | | 1 | | 0 | | 1 | | 2 | 1 | | 0 | | 9 | | 3 | |  |
| Al-Hakmani, FM. et al., 2016 [110] | 1 | 1 | 1 | 1 | 3 | 1 | 1 | 1 | | 1 | | 0 | | 1 | | 2 | 1 | | 0 | | 10 | | 2 | |  |
| Alhainiah, MH. et al., 2018 [49] | 1 | 1 | 1 | 1 | 0 | 1 | 1 | 1 | | 1 | | 3 | | 1 | | 2 | 1 | | 0 | | 10 | | 2 | |  |
| Alsammani, MA.  Ahmed, SR. 2015 [69] | 1 | 1 | 1 | 1 | 0 | 1 | 1 | 1 | | 1 | | 3 | | 1 | | 2 | 1 | | 0 | | 10 | | 2 | |  |
| Shawky, S. and Milaat, W. 2000 [90] | 1 | 1 | 1 | 1 | 0 | 1 | 1 | 1 | | 1 | | 3 | | 1 | | 2 | 1 | | 1 | | 11 | | 1 | |  |
| Al Rowaily, MA. Et al., 2014 [74] | 1 | 1 | 1 | 1 | 0 | 1 | 1 | 1 | | 1 | | 2 | | 1 | | 2 | 1 | | 1 | | 11 | | 1 | |  |
| El Mallah, KO. Et al., 1997 [92] | 1 | 1 | 1 | 1 | 0 | 1 | 1 | 1 | | 1 | | 2 | | 1 | | 2 | 1 | | 0 | | 10 | | 2 | |  |
| Barakat, MN. et al., [113] | 1 | 1 | 1 | 1 | 0 | 1 | 1 | 1 | | 1 | | 2 | | 1 | | 2 | 1 | | 1 | | 11 | | 1 | |  |
| Mansouri, HA. and  Ghazawi, AH. 2007 [85] | 1 | 1 | 1 | 1 | 0 | 1 | 1 | 1 | | 1 | | 2 | | 1 | | 2 | 1 | | 0 | | 10 | | 2 | |  |
| Abdalrahman Almarzouki, A. 2003 [88] | 1 | 1 | 1 | 1 | 0 | 1 | 1 | 1 | | 1 | | 2 | | 1 | | 2 | 1 | | 0 | | 10 | | 2 | |  |
| Al-Hakeem, MM. 2006 [86] | 1 | 1 | 1 | 1 | 0 | 1 | 1 | 1 | | 1 | | 2 | | 1 | | 2 | 1 | | 0 | | 10 | | 2 | |  |
| Yamani Zamzami, TY., 2004 [87] | 1 | 1 | 1 | 1 | 0 | 1 | 1 | 1 | | 1 | | 2 | | 1 | | 2 | 1 | | 0 | | 10 | | 2 | |  |
| Makhseed, M. et al., 1994 [48] | 1 | 1 | 3 | 1 | 0 | 1 | 1 | 1 | | 1 | | 3 | | 1 | | 2 | 3 | | 0 | | 8 | | 2 | |  |
| Al-Farsi, YM. et al., 2011 [112] | 1 | 1 | 1 | 1 | 0 | 1 | 1 | 1 | | 0 | | 3 | | 1 | | 4 | 2 | | 1 | | 9 | | 2 | |  |
| Wahabi, HA. et al., 2017 [62] | 1 | 1 | 1 | 1 | 1 | 1 | 1 | 1 | | 1 | | 3 | | 1 | | 4 | 4 | | 1 | | 11 | | 0 | |  |
| Almakoshi, A. et al., 2015 [70] | 1 | 1 | 4 | 1 | 0 | 1 | 2 | 1 | | 1 | | 1 | | 1 | | 4 | 1 | | 1 | | 10 | | 1 | |  |
| Wahabi, HA. et al., 2014 [72] | 1 | 1 | 1 | 1 | 1 | 3 | 1 | 1 | | 1 | | 3 | | 1 | | 4 | 3 | | 1 | | 10 | | 0 | |  |
| Wahabi, HA. et al., 2013 [75] | 1 | 1 | 1 | 1 | 1 | 1 | 1 | 1 | | 1 | | 3 | | 1 | | 4 | 3 | | 1 | | 11 | | 0 | |  |
| Wahabi, HA et al., 2013 [76] | 1 | 1 | 1 | 1 | 1 | 1 | 1 | 0 | | 1 | | 3 | | 1 | | 4 | 4 | | 1 | | 10 | | 1 | |  |
| Wahabi, HA et al., 2013 [77] | 1 | 1 | 1 | 1 | 1 | 1 | 1 | 1 | | 1 | | 3 | | 1 | | 4 | 1 | | 0 | | 11 | | 1 | |  |
| Wahabi, HA et al., 2012 [26] | 1 | 1 | 1 | 1 | 1 | 3 | 1 | 1 | | 1 | | 3 | | 1 | | 1 | 3 | | 1 | | 11 | | 0 | |  |
| Al-Sunaidi MI and Al-Shabrani MS, 2011 [81] | 1 | 1 | 2 | 1 | 0 | 1 | 1 | 3 | | 1 | | 3 | | 1 | | 1 | 2 | | 0 | | 8 | | 2 | |  |
| Al Arfaj AS and Khalil N, 2010 [27] | 1 | 1 | 2 | 1 | 1 | 1 | 2 | 1 | | 1 | | 3 | | 1 | | 4 | 3 | | 1 | | 10 | | 0 | |  |
| Al-Mulhim, A. et al., 2003 [25] | 1 | 1 | 3 | 1 | 0 | 1 | 1 | 1 | | 1 | | 3 | | 1 | | 2 | 3 | | 0 | | 8 | | 2 | |  |
| Al-Ali, FM. et al., 1997 [107] | 1 | 1 | 1 | 1 | 1 | 1 | 1 | 1 | | 1 | | 1 | | 1 | | 4 | 4 | | 1 | | 11 | | 0 | |  |
| Fayed, HM. et al., 1993 [93] | 1 | 1 | 1 | 1 | 1 | 1 | 1 | 1 | | 1 | | 2 | | 1 | | 0 | 1 | | 0 | | 11 | | 2 | |  |
| *Mean number of criteria with “potentially low ROB”* | *–* | *–* | *–* | *–* | *–* | *–* | *–* | *–* | *–* | | *–* | | *–* | | *–* | | | *–* | | *–* | | *9.9* | | *1.8* | |
| Overall mean number of criteria | – | – | – | – | – | – | – | – | – | | – | | – | | – | | | – | | – | | 9.8 | | 1.6 | |

ROB: risk of bias; 1: Yes (potential low ROB); 0: No (potential high ROB); 2: can’t determine; 3: not applicable; 4: not reported.
