# Supplementary material for: Maternal and birth cohort studies in the Gulf Cooperation Council countries: a systematic review and meta-analysis
Source: Syst Rev. 2020 Jan 16;9:14. doi: 10.1186/s13643-020-1277-0 (PMC6964097; doi:10.1186/s13643-020-1277-0)
Supplement: Supplementary file 6 [file 13643_2020_1277_MOESM6_ESM.docx]

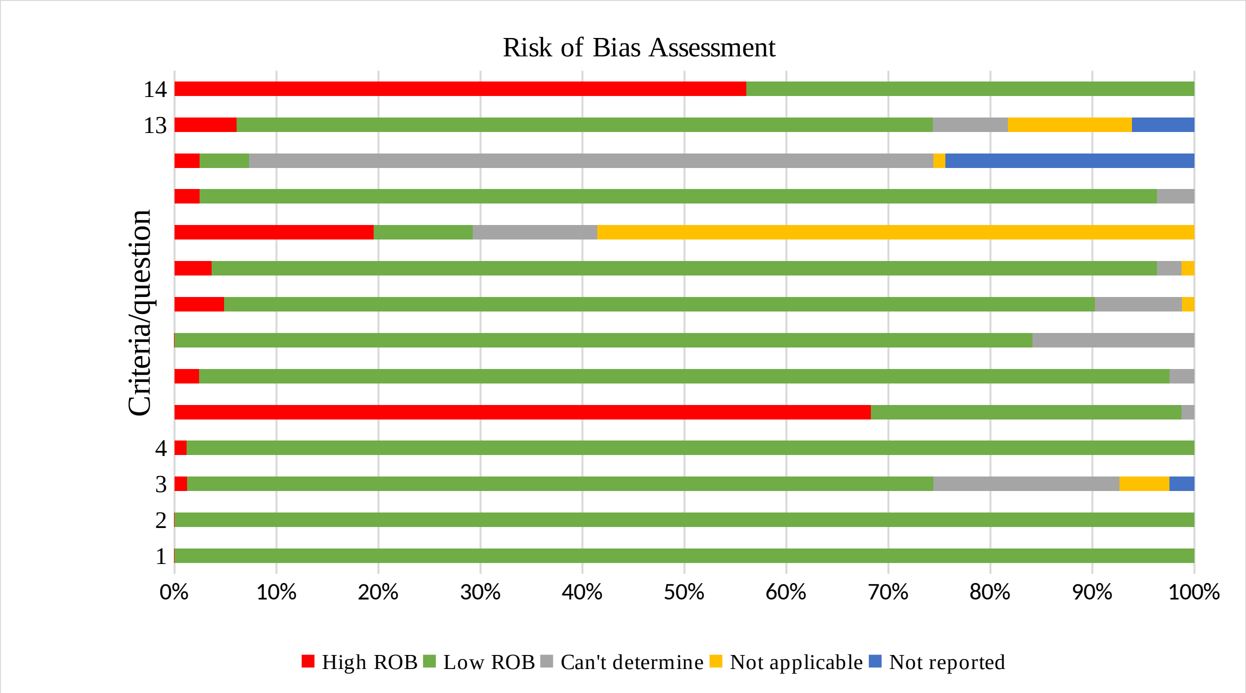

Risk of Bias Assessment
Criteria/question
14
13
4
3
2
1
0%
10%
20%
30%
40%
50%
60%
70%
80%
90%
100%
High ROB
Low ROB
Can't determine
Not applicable
Not reported
